# Effect of an Enhanced Paramedic Acute Stroke Treatment Assessment on Thrombolysis Delivery During Emergency Stroke Care: A Cluster Randomized Clinical Trial

**DOI:** 10.1001/jamaneurol.2020.0611

**Published:** 2020-04-13

**Authors:** Christopher I. Price, Lisa Shaw, Saiful Islam, Mehdi Javanbakht, Alan Watkins, Peter McMeekin, Helen Snooks, Darren Flynn, Richard Francis, Rachel Lakey, Lou Sutcliffe, Graham McClelland, Joanne Lally, Catherine Exley, Helen Rodgers, Ian Russell, Luke Vale, Gary A. Ford

**Affiliations:** 1 Stroke Research Group, Institute of Neuroscience, Newcastle University, Newcastle upon Tyne, United Kingdom; 2 Swansea University Medical School, Swansea, Wales, United Kingdom; 3 Institute of Health and Society, Newcastle University, Newcastle upon Tyne, United Kingdom; 4 Faculty of Health and Life Sciences, Northumbria University, Newcastle upon Tyne, United Kingdom; 5 Newcastle Clinical Trials Unit, Newcastle University, Newcastle upon Tyne, United Kingdom; 6 North East Ambulance Service, Newcastle upon Tyne, United Kingdom; 7 Newcastle upon Tyne Hospitals National Health Service Foundation Trust, Newcastle upon Tyne, United Kingdom; 8 Medical Sciences Division, University of Oxford, Oxford, United Kingdom; 9 Oxford University Hospitals National Health Service Foundation Trust, Oxford, United Kingdom

## Abstract

**Importance:**

Rapid thrombolysis treatment for acute ischemic stroke reduces disability among patients who are carefully selected, but service delivery is challenging.

**Objective:**

To determine whether an enhanced Paramedic Acute Stroke Treatment Assessment (PASTA) intervention increased hospital thrombolysis rates.

**Design, Setting, and Participants:**

This multicenter, cluster randomized clinical trial took place between December 2015 and July 2018 in 3 ambulance services and 15 hospitals. Clusters were paramedics based within ambulance stations prerandomized to PASTA or standard care. Patients attended by study paramedics were enrolled after admission if a hospital specialist confirmed a stroke and paramedic assessment started within 4 hours of onset. Allocation to PASTA or standard care reflected the attending paramedic’s randomization status.

**Interventions:**

The PASTA intervention included additional prehospital information collection, a structured hospital handover, practical assistance up to 15 minutes after handover, a predeparture care checklist, and clinician feedback. Standard care reflected national guidelines.

**Main Outcomes and Measures:**

Primary outcome was the proportion of patients receiving thrombolysis. Secondary outcomes included time intervals and day 90 health (with poor status defined as a modified Rankin Score >2, to represent dependency or death).

**Results:**

A total of 11 478 patients were screened following ambulance transportation; 1391 were eligible and approached, but 177 did not consent. Of 1214 patients enrolled (mean [SD] age, 74.7 [13.2] years; 590 women [48.6%]), 500 were assessed by 242 paramedics trained in the PASTA intervention and 714 were assessed by 355 paramedics continuing with standard care. The paramedics trained in the PASTA intervention took a mean of 13.4 (95% CI, 9.4-17.4) minutes longer (*P* < .001) to complete patient care episodes. There was less thrombolysis among the patients in the PASTA group, but this was not significant (PASTA group, 197 of 500 patients [39.4%] vs the standard care group, 319 of 714 patients [44.7%]; adjusted odds ratio, 0.81 [95% CI, 0.61-1.08]; *P* = .15). Time from a paramedic on scene to thrombolysis was a mean of 8.5 minutes longer in the PASTA group (98.1 [37.6] minutes) vs the standard care group (89.4 [31.1] minutes; *P* = .01). Poor health outcomes did not differ significantly but occurred less often among patients in the PASTA group (313 of 489 patients [64.0%]) vs the standard care group (461 of 690 patients [66.8%]; adjusted odds ratio, 0.86 [95% CI, 0.60-1.20]; *P* = .39).

**Conclusions and Relevance:**

An enhanced paramedic assessment did not facilitate thrombolysis delivery. The unexpected combination of thrombolysis and health outcomes suggests possible alternative influences on treatment decisions by the intervention, requiring further evaluation.

**Trial Registration:**

ISRCTN Registry Identifier: ISRCTN12418919

## Key Points

**Question:**

Can hospital stroke thrombolysis treatment rates be increased by an enhanced paramedic assessment that includes additional prehospital information collection, a structured hospital handover, practical assistance after handover, a predeparture care checklist, and clinician feedback?

**Findings:**

In this cluster randomized clinical trial, fewer patients in the intervention group (39.4%) received thrombolysis vs those in the standard care group (44.7%), but there were fewer poor health outcomes (disability or death) after 90 days (intervention group, 64.0% vs standard care group, 66.8%). The results were not statistically significant.

**Meaning:**

This study found that the enhanced paramedic assessment should not be used to increase thrombolysis volume but may influence the quality of treatment decisions.

## Teaser

This cluster randomized clinical trial sought to examine whether adding enhanced paramedic assessment to standard care would result in increased rates of thrombolysis in patients experiencing stroke.

## Introduction

Intravenous thrombolysis administered within 4.5 hours of symptom onset reduces disability following acute ischemic stroke, but outcomes are highly time dependent.^1^ In the UK, national audit reports continue to show large differences in performance between services and diurnal fluctuations attributable to variations in local assessment processes and stroke specialist availability.^2,3,4^ Improvements in delivery have resulted from hospital-based reorganization of services,^4,5,6^ but many sites still report suboptimal rates and slow treatment times.^2,3^

The prehospital phase of emergency stroke care has been targeted with interventions to facilitate thrombolysis treatment, including multiprofessional workforce training,^6^ raising the service priority level for suspected stroke,^7^ hospital prenotification,^8^ and personalized feedback to paramedics about care quality.^9^ Reports have described short-term improvements, but studies were setting specific and/or observational. In other specialties, there is increasing evidence that simple tools can standardize communication of key information and confirm whether essential tasks have been undertaken, including structured formats for paramedic handover to emergency department staff^10,11^ and multidisciplinary care process checklists.^12,13^ It has previously been demonstrated that key information for emergency stroke treatment decisions is often missing from ambulance clinical records.^14^ Enhanced handover and team checklists might therefore be valuable during the routine assessment of thrombolysis eligibility. We examined whether it was possible to improve thrombolysis delivery in hospital following introduction of an enhanced Paramedic Acute Stroke Treatment Assessment (PASTA) intervention without additional hospital-based reorganization or whole-system modification.

## Methods

### Study Design

A pragmatic, multicenter cluster randomized clinical trial design was chosen to reduce contamination of standard care by the intervention and avoid any potential delays in care associated with individual randomization. Ethical approval was granted by the National Research Ethics Committee North East–Newcastle and North Tyneside 1. The detailed study protocol (Supplement 2) has been published.^15^

### Study Setting

The study was hosted by 3 ambulance services (North East England, North West England, and Wales) and 15 hospitals with acute stroke units (the equivalent to 4 comprehensive and 11 primary centers). Each ambulance service had similar clinical pathways for standard care (SC) that reflected national clinical guidelines,^16^ including identification using the Face Arm Speech Test,^17^ exclusion of hypoglycemia, and urgent transportation with prenotification to the nearest hospital when time since symptom onset was less than 4 hours. Participating hospital characteristics are shown in eTable 1 in Supplement 1.

### Randomization and Blinding

Clusters were the individual paramedics based within prerandomized ambulance stations stratified by service, size, and distance of station from the nearest study hospital. All ambulance stations serving a study hospital were allocated to PASTA or SC using simple randomization by the trial statistician (S.I.). Paramedics based at stations randomized to PASTA only became involved after successful completion of study-specific training (an online video and a knowledge assessment). Paramedics based at SC stations were simply informed that their clinical record entries would be supporting a study of prehospital assessment. Patients received PASTA or SC according to which paramedic attended the incident. Participating hospitals were not randomized and received patients from both the PASTA and SC groups. Because of the nature of the intervention, blinding was not possible.

### Participants

Patients were identified and recruited after completion of the thrombolysis assessment in participating hospitals. Adult patients with stroke were eligible for inclusion if a hospital specialist confirmed a stroke diagnosis and a study paramedic had attended within 4 hours of symptom onset.

Patients with capacity gave written informed consent. If capacity was absent, enrollment was sought via a personal or a professional consultee. If the patient died before consent could be obtained, the local principal investigator completed an early mortality declaration form designed specifically for this study, and their data were included in the analysis. Postal consent was introduced following a protocol amendment as an additional method for patients who were discharged early.

### Interventions

**Figure 1.**
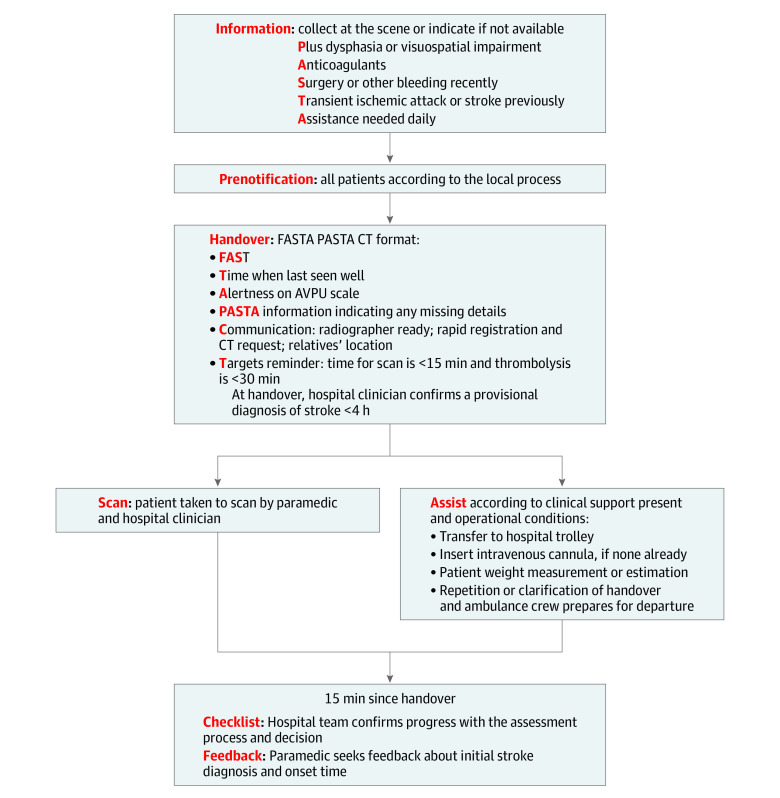
Paramedic Acute Stroke Treatment Assessment (PASTA) Pathway Diagram AVPU indicates alert, verbally responsive, responsive to pain, or unresponsive; CT, communication and targets; FAST, Face Arm Speech Test.

A summary of the PASTA pathway is shown in Figure 1 and a detailed description is given in the eMethods in Supplement 1. The content had been developed over 12 months prior to study launch using previously published reports and interactive workshops with personnel from all participating ambulance services and hospitals. Key features were additional prehospital data collection aimed at thrombolysis treatment criteria (ie, symptom severity, medical history, clarification of onset time), a corresponding structured hospital handover, assisting with simple care tasks in hospital for up to 15 minutes including transfer to the scan room, completing a basic thrombolysis assessment checklist before departure, and seeking clinician feedback. Trained paramedics were requested to provide the intervention to patients whom they suspected were suffering a stroke and were within 4 hours of symptom onset. Initial paramedic stroke identification processes were unchanged. A study-specific ambulance data collection form was completed to record delivery of the different PASTA components. Patients attended by paramedics randomized to SC received routine assessment and treatment according to national clinical guidelines.^16^

### Outcome Measures

The primary outcome was the proportion of patients receiving thrombolysis. Secondary outcomes included key time intervals during assessment and thrombolysis treatment, stroke severity 24 hours after thrombolysis (National Institute of Health Stroke Scale^18^ [NIHSS]), complications after thrombolysis, delivery of other components of acute care, day 90 dependency (modified Rankin Scale [mRS] score^19,20^) and day 90 mortality. Complications assessed included Symptomatic Intracranial Hemorrhage, which was defined as at least 4 points of deterioration on the 24-hour NIHSS score with evidence of any hemorrhage on repeated brain imaging.^21^ A standard definition was used for serious adverse events, but reporting was not required for preplanned hospitalizations and treatments for preexisting conditions and predefined expected adverse events. Outcome data were collected by hospital research staff from routinely available records and 1 study-specific assessment (undertaken by telephone, mail, or face-to-face contact) at day 90 post stroke.

The primary outcome originally registered was the day 90 mRS score. However, as described in the published protocol, this was formally amended in October 2017 to the proportion of patients receiving thrombolysis.^15^

### Statistical Analysis

Based on outcomes reported by previous studies and our eligibility criteria, the sample size estimation considered that a change from 43% to 53% of patients eligible for the study receiving thrombolysis would be clinically important. At 90% power, 5% significance, a mean cluster size (patients per paramedic) of 5, an intracluster correlation coefficient of 0.02, an imbalance of 2 control patients per intervention patient (reflecting delays in PASTA training uptake), and an attrition rate of 1%, it was calculated that 1297 patients were required (865 in the control group and 432 in the intervention group). However, the study protocol allowed for the final recruitment target to be kept under review and adjusted to reflect any changes in the underlying assumptions. The final required number of patients was 1149, based on a cluster size of 3 and a control:intervention imbalance of 1.6.

Analysis was by treatment allocated (the study group allocation of the station base for the attending paramedic). Study paramedics did not change their PASTA or SC allocation if they changed stations during the trial. Imputation was used for missing NIHSS and day 90 mRS scores.

The primary analysis used logistic regression allowing for clustering by paramedic, with adjustment for clinically important and statistically significant covariates and factors to estimate an adjusted odds ratio (aOR) for the proportion of all patients receiving thrombolysis. The adjustment variables were not prespecified but determined by stepwise backward selection from a master list of potential covariates. A Cox regression estimated a hazard ratio for the combined outcome of the intervention on thrombolysis and time to treatment since the emergency call. Other time intervals were compared by *t* tests after excluding prespecified improbable time intervals. The NIHSS scores were compared using *t* tests. Odds ratios were calculated using logistic regression for all other comparisons. Additional binary variables were derived from relevant outcomes to indicate if patients had any complication after thrombolysis and received 1 or more acute care interventions, and ORs were calculated. The mRS was dichotomized into favorable outcome (mRS, 0-2) or poor outcome (mRS, 3-6) and an aOR of a poor outcome calculated, adjusting as above. Statistical significance was defined by *P* ≤ .05, 2 tailed. Analyses were carried out using SPSS version 25 (IBM).

A post hoc analysis considered whether routine hospital specialist availability for thrombolysis decision-making had any bearing on treatment received in each study group (as per an odds ratio calculation). Workforce information reported in the National Sentinel Stroke Audit Programme Acute Organisational Audit 2016^2^ was used to categorize hospitals as compliant or noncompliant with the current national recommendation regarding hospital provision of a specialist thrombolysis service (ie, there should be a minimum of 6 specialists trained in emergency stroke care providing a continuous rota without input from nonspecialists, so that all treatment decisions are made by a stroke specialist) (eTable 1 in Supplement 1).^16^

## Results

**Figure 2.**
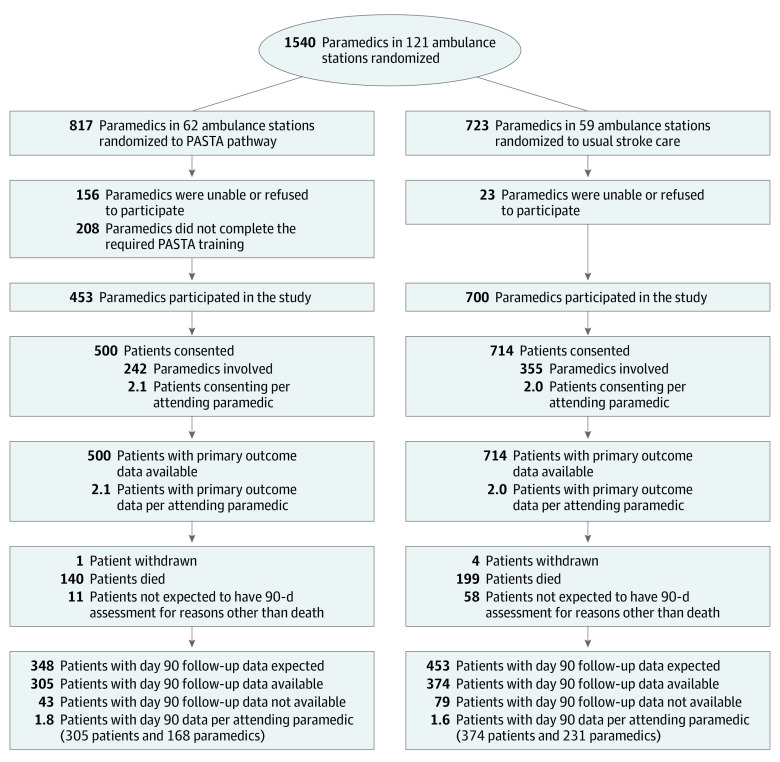
Trial Diagram Seven hundred paramedics participated in the study while continuing standard care, of whom 8 completed PASTA training during the course of the study. PASTA indicates Paramedic Acute Stroke Treatment Assessment.

One hundred twenty-one ambulance stations with 1540 paramedics were involved in the trial. At 62 PASTA stations, 453 of 817 paramedics (55.4%) completed training. At 59 SC stations, 700 of 723 paramedics (96.8%) agreed to assist. Between December 10, 2015, and July 31, 2018, 11 478 patients with stroke who traveled by ambulance were screened, 1391 fulfilled the eligibility criteria and were approached, and 1214 patients were enrolled. Of these, 500 were assessed by 242 paramedics trained in the PASTA intervention (2.1 patients/paramedic) and 714 by 355 paramedics continuing with SC (2.0 patients/paramedic). Follow-up is shown in Figure 2. Primary outcome data were available for all patients.

Demographic and clinical characteristics were similar in the 2 study groups for all patients (Table 1) and for patients treated with thrombolysis (eTable 3 in Supplement 1). The mean (SD) age was 74.7 (13.2) years; 590 of 1214 patients (48.6%) were women, and the median and mean admission NIHSS scores were 9.0 and 11.4, respectively.

**Table 1.** Demography and Clinical Characteristics

Characteristic	PASTA	Standard care
Total	500	714
Sex, No. (%)		
Male	259 (51.8)	365 (51.1)
Female	241 (48.2)	349 (48.9)
Age, median (interquartile range), y	76.5 (68.0-84.0)	77.0 (67.8-84.0)
Prestroke modified Rankin scale score, No. (%)		
Patients with data available	494 (100)	708 (100)
0	233 (47.2)	341 (48.2)
1	78 (15.8)	126 (17.8)
2	65 (13.2)	79 (11.2)
3	66 (13.4)	97 (13.7)
4	42 (8.5)	47 (6.6)
5	10 (2.0)	18 (2.5)
0-2	376 (76.1)	546 (77.1)
3-5	118 (23.9)	162 (22.9)
Stroke severity at admission, NIHSS score		
Patients with data available	499 (100)	710 (100)
Median (interquartile range)	8 (4-17)	9 (4-19)
Mean (SD)	11.1 (8.7)	11.5 (8.5)
Results of the first brain imaging, No. (%)		
Patients with data available	499 (100)	714 (100)
Infarction	409 (82.0)	607 (85.0)
Primary intracerebral hemorrhage	90 (18.0)	106 (14.8)
Other	0	1 (0.1)
Blood pressure on admission		
Systolic		
Patients with data available, No.	497	712
Median (interquartile range)	158 (136-178)	158 (138-175)
Mean (SD)	158.4 (30.8)	157.4 (29.8)
Diastolic		
Patients with data available, No.	497	712
Median (interquartile range)	85 (73-97)	82 (71-94)
Mean (SD)	85.2 (19.0)	83.9 (18.2)
Blood glucose on admission		
Patients with data available, No.	478	682
Median (interquartile range)	6.7 (5.7-7.9)	6.7 (5.7-8.1)
Mean (SD)	7.4 (2.8)	7.4 (3.2)
Anticoagulation on admission, No. (%)		
Patients with data available	499 (100)	713 (100)
Warfarin	42 (8.4)	51 (7.2)
Apixaban	9 (1.8)	24 (3.4)
Rivaroxaban	12 (2.4)	22 (3.1)
Dabigatran	3 (0.6)	2 (0.3)

Abbreviation: PASTA, Paramedic Acute Stroke Treatment Assessment.

Following the 999 call, paramedics trained in the PASTA intervention took a mean of 13.4 (95% CI, 9.4-17.4) minutes longer (*P* < .001) to complete (or clear) patient care episodes than paramedics continuing with SC, mainly because an additional 8.8 (95% CI, 6.5-11.0) additional minutes (*P* < .001) were spent in the hospital. There was no significant difference between the groups for paramedic time spent on the scene (PASTA group, 26.0 minutes; SC group, 24.2 minutes; difference, 1.61 [95% CI, −0.2 to 3.4] minutes; *P* = .08). Other key ambulance time intervals are shown in eTable 4 in Supplement 1.

Details about thrombolysis treatment are shown in Table 2. There was no significant difference in the proportion of patients who received thrombolysis (primary outcome) between PASTA (197 of 500 patients [39.4%]) and SC (319 of 714 [44.7%]), but the general direction of this finding was the opposite of the anticipated outcome of the intervention (adjusted odds ratio, 0.81 [95% CI, 0.61-1.08]; *P* = .15; intraclass correlation coefficient, 0.00). The pattern among patients with ischemic stroke was similar.

**Table 2.** Thrombolysis Treatment

Outcome	Patients in PASTA group, No. (%)	Patients in SC group, No. (%)	Odds ratios (95% CI) or difference in mean PASTA − standard care outcomes (95% CI)	*P* value
Thrombolysis treatment				
All patients				
Unadjusted odds ratio (95% CI)	197^a^ (39.4)	319^a^ (44.7)	0.81 (0.64-1.02)	.07
Adjusted odds ratio (95% CI)			0.81 (0.61-1.08)	.15
Ischemic stroke only				
Unadjusted odds ratio (95% CI)	196^b^^,^^c^ (47.9)	319^b^ (52.6)	0.83 (0.65-1.07)	.15
Adjusted odds ratio (95% CI)			0.84 (0.60-1.17)	.30
8 am to 8 pm				
Unadjusted odds ratio (95% CI)	130^d^ (39.0)	208^d^ (45.2)	0.78 (0.58-1.03)	.08
Adjusted odds ratio (95% CI)			0.80 (0.55-1.15)	.23
8 pm to 8 am				
Unadjusted odds ratio (95% CI)	46^e^ (38.0)	79^e^ (41.4)	0.87 (0.55-1.39)	.56
Adjusted odds ratio (95% CI)			0.85 (0.46-1.56)	.59
Complications after thrombolysis, No. (%)				
Patients, No.	196	316	NA	NA
Symptomatic intracranial hemorrhage	4 (2.0)	10 (3.1)	0.64 (0.20-2.10)	.46
Extracranial hemorrhage	6 (3.1)	6 (1.9)	1.65 (0.52-5.20)	.39
Angioedema	2 (1.0)	7 (2.2)	0.46 (0.10-2.24)	.32
Other complication	2 (1.0)	1 (0.3)	3.3 (0.30-36.40)	.56
Any complication	13 (6.6)	24 (7.5)	0.87 (0.43-1.76)	.70
Stroke onset to thrombolysis treatment time, min				
Patients, No.	197	319	NA	NA
Mean (SD)	154.4 (55.3)	149.9 (51.7)	4.47 (−4.97 to 13.93)^f^	.35
Median (interquartile range)	146 (110-194)	137 (110-190)	NA	NA
Paramedic assessment to thrombolysis treatment time, min				
Patients, No.	194	315	NA	NA
Mean (SD)	98.1 (37.6)	89.6 (31.1)	8.50 (2.10-14.80)^f^	.01
Median (interquartile range)	90 (72-114)	86 (68-107)	NA	NA
Hospital arrival to thrombolysis treatment time, min				
Patients, No.	176	286	NA	NA
Mean (SD)	58.9 (33.4)	54.2 (26.9)	4.69 (−1.20 to 10.55)^f^	.12
Median (interquartile range)	48.5 (35-75)	48.5 (36-65)	NA	NA
Stroke severity^g^				
After thrombolysis treatment (24-48 h)				
Patients, No.	193	307	NA	NA
Mean (SD)	8.5 (9.0)	9.6 (9.3)	−1.12 (−2.7 to 0.54)^f^	.19
Median (interquartile range)	5 (1-14)	6 (2-15)	NA	NA
Reduction after thrombolysis treatment				
Patients, No.	193	307	NA	NA
Mean (SD)	3.7 (6.5)	2.8 (7.2)	0.90 (−0.35 to 2.2)^f^	.16
Median (interquartile range)	4 (0-7)	3 (0-7)	NA	NA

Abbreviations: OR, odds ratio; PASTA, Paramedic Acute Stroke Treatment Assessment; SC, standard care group.

^a^ PASTA group, n = 500; SC group, n = 714.

^b^ PASTA group, n = 409; SC group, n = 607.

^c^ This is 196 patients, not 197, because 1 patient with a subtle hemorrhagic stroke who was not initially identified on the admission computed tomography scan received thrombolysis.

^d^ PASTA group, n = 333; SC group, n = 460.

^e^ PASTA group, n = 121; SC group, n = 191.

^f^ Difference in mean PASTA and standard care outcome.

^g^ National Institutes of Health Stroke Scale score.

In patients treated with thrombolysis, paramedic assessment-to-thrombolysis time in the PASTA group was longer by a mean of 8.5 minutes (95% CI, 2.1-13.9 minutes; *P* = .01) compared with the SC group. Smaller differences in door-to-needle and onset-to-needle times did not reach statistical significance, presumably reflecting influences external to the study intervention (ie, public response to symptoms and ambulance availability) and reduced statistical power. The Cox regression analysis of time from 999 call to treatment for intervention versus control groups reported an adjusted hazard ratio of 0.85 (95% CI, 0.71-1.02; coefficient, −0.17; *P* = .07), indicating that intervention group thrombolysis was less likely at any point after the start of the emergency care pathway, although statistical significance was not reached. After thrombolysis, there were no significant differences between groups for reduction in stroke severity or any treatment complication, but the number of events was small (Table 2).

No significant differences were observed for other individual acute care processes delivered to all patients (eTable 5 in Supplement 1) or in the number of patients in the PASTA group who received at least 1 acute care component relative to SC (odds ratio, 1.16 [95% CI, 0.88-1.54]; *P* = .28). There was no significant difference between groups for time from stroke onset to first brain scan.

**Figure 3.**
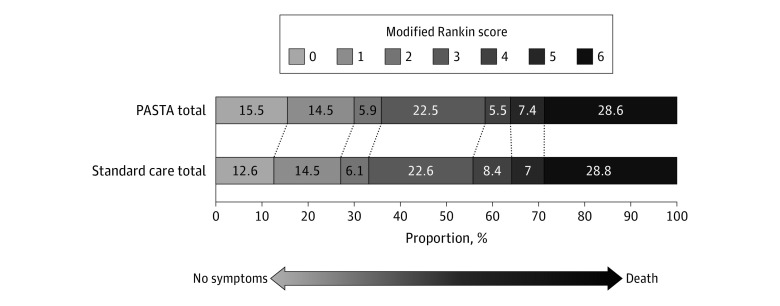
Distribution of Modified Rankin Scores at Day 90 PASTA indicates Paramedic Acute Stroke Treatment Assessment.

At day 90, there was no significant difference between groups for mortality (PASTA, 140 of 499 patients [28.1%] vs SC, 199 of 712 patients [27.9%]; odds ratio, 1.00 [95% CI, 0.78-1.30]; *P* = .97). Figure 3 shows the distribution of mRS values at day 90. Although lacking statistical significance, there were numerically fewer poor outcomes (mRS score ≥3) at day 90 among patients in the intervention group (PASTA, 313 of 489 patients [64.0%] vs SC, 461 of 690 patients [66.8%]; aOR, 0.86 [95% CI, 0.60-1.2]; *P* = .39), which was also seen among those who received thrombolysis (PASTA, 108 of 193 patients [56.0%] vs 191 of 312 patients [61.2%]; aOR, 0.78 [95% CI, 0.47-1.30]; *P* = .34). eFigures 1 and 2 in Supplement 1 show day 90 mRS distributions for thrombolysis and nonthrombolysis groups. Serious adverse events were recorded from 81 patients in the PASTA group (16.2%; 94 total events) and 136 patients in the SC group (19%; 161 total events). None had a causal link to the study intervention.

In the post hoc analysis, 8 hospitals were not fully compliant with the national recommendation for local specialist availability (eTable 1 in Supplement 1). These noncompliant services had a statistically significant 9.8% absolute reduction in the PASTA thrombolysis treatment rate relative to SC (PASTA, 99 of 276 patients [35.9%] vs SC, 105 of 230 patients [45.7%]; unadjusted OR, 0.67 [95% CI, 0.47-0.95]; *P* = .03), whereas the 7 hospitals that were compliant showed only a 0.4% reduction (PASTA, 98 of 224 patients [43.8%] vs SC, 214 of 484 patients [44.2%]; unadjusted OR, 0.98 [95% CI, 0.71-1.35]; *P* = .91).

Study-specific ambulance data collection forms recording delivery of the PASTA pathway were located for 227 of 500 patients (45.5%) in the intervention group. Use of the structured handover was recorded for 134 of 227 patients (59.0%). Full data are shown in eTable 6 in Supplement 1.

## Discussion

This multisite pragmatic trial showed that a paramedic-led thrombolysis-focused emergency stroke assessment that extended beyond hospital handover did not increase thrombolysis rates. Instead, a nonsignificant reduction in thrombolysis administration was observed. Although there was a longer initial paramedic assessment process, it is unlikely that the PASTA intervention resulted in patients simply timing out of treatment, because this was proportionally a minor extension of the whole emergency pathway, and the Cox regression analysis indicated that intervention thrombolysis was less likely at any point since the emergency call.

It may be surprising that the PASTA pathway did not improve thrombolysis delivery when simpler prehospital interventions have increased treatment rates (eg, raising the ambulance priority level for suspected stroke^7^) and reduced hospital treatment delays (eg, prenotification^8^), but the service context of each report is likely to be relevant. Previously, additional thrombolysis activity was observed at 4 of 6 US centers following a multilevel intervention including public awareness activities, a paramedic symptom checklist, and competitive benchmarking.^6^ The 2 unchanged centers had high baseline treatment rates and may have already achieved optimal performance. A similar ceiling effect may explain the lack of effect among PASTA sites, which were already established thrombolysis providers. A multisite Scandinavian trial^7^ randomized 942 patients with suspected stroke or transient ischemic attack to a higher vs a standard response level after multidisciplinary training and reported a thrombolysis rate of 24% compared with 10% among the control group. As with PASTA, there was no significant change in door-to-needle time, suggesting that delays following admission are more likely to reflect logistical factors, such as scan capacity, image reporting, and specialist availability. In our results, the relatively long mean door-to-needle time in standard care of 54 minutes may indicate services that lacked the logistical capacity to improve. It is important to recognize that PASTA did not change public awareness, priority response level, identification, or prenotification. Because UK standard care already includes these components, incremental gains from new interventions become harder to achieve, and an enhanced paramedic assessment may not be powerful enough to augment treatment delivery in contemporary thrombolysis-active services.

Despite the intervention group showing a surprising nonsignificant finding of fewer thrombolysis treatments, outcomes were not adversely affected and there was a counterintuitive nonsignificant result of better health at day 90. It is important to acknowledge that statistical power for any secondary outcome was lacking, and such differences between groups may simply reflect chance. However, if indicative of a genuine effect, one possible hypothesis for this apparent paradox is an influence on case selection; ie, structured communication of directly relevant and timely information by PASTA paramedics might increase clinician confidence about withholding treatment when there is borderline benefit, higher-than-typical risk, or uncertainty about key details, such as onset time. The post hoc analysis showed that intervention-group thrombolysis was significantly less likely across services with specialist availability below the level recommended by national guidelines. Relatively inexperienced clinicians under time pressure may tend toward overtreatment rather than undertreatment of patients with borderline cases, which could be moderated by the PASTA handover and/or checklist, whereas services with greater specialist continuity may already apply a more systematic approach to case selection. Because this was an unexpected finding, we had not collected the required data describing clinical and radiological quality of individual treatment decisions to confirm this hypothesis, and therefore further research is needed. However, previous emergency department studies have reported that typically, less than half of pertinent data points are shared during standard handover of mixed patient groups,^22^ with significant variation attributable to clinician experience.^11,23^ The relevance and clarity of handover can be improved by introduction of simple generic formats,^10,24^ while multidisciplinary team checklists make care safer through clarification of information and reinforcement of important standards.^12,13^

### Limitations

A key limitation of the study was the inability to mask group allocation from clinicians because of the nature of the intervention, but the lack of imbalance in baseline characteristics makes selection bias an unlikely explanation for the results. Better health outcomes might also result if the intervention favored labelling less disabling mimic conditions as stroke, but this seems unlikely, because patients were only enrolled after local specialist confirmation of a stroke diagnosis. Challenging operational conditions impeded objective confirmation of intervention fidelity, and approximately half of the study ambulance data forms were not returned, despite efforts to encourage completion. However, since the mean additional time spent by PASTA paramedics on each call was nearly 15 minutes, and all had completed the study training, it is probable that some aspects of the intervention were being performed. As a pragmatic real-world evaluation across multiple sites with an inclusive consent strategy, the result remains relevant for modern clinical services.

## Conclusions

The PASTA trial showed that incremental improvements in thrombolysis delivery are unlikely to be achieved through isolated use of more sophisticated prehospital assessments. An unexpected finding led us to consider whether structured handover of additional information and/or a multidisciplinary checklist could improve the selection of patients for thrombolysis, particularly in hospitals with lower levels of specialist availability. Further research is required to confirm this hypothesis and demonstrate whether the intervention promotes quality rather than quantity during emergency stroke care.

## Notes

Supplement 1. **eTable 1.** Participating hospital site characteristics. **eMethods.** Detailed description of the PASTA pathway. **eTable 2.** Permitted local variations to the PASTA pathway. **eTable 3.** Demography and clinical characteristics according to study group and receipt of IV thrombolysis. **eTable 4.** Key ambulance time intervals. **eTable 5.** Other acute care in hospital and key hospital time intervals. **eTable 6.** Full details recorded about PASTA pathway delivery. **eFigure 1.** Distribution of mRS scores at day 90 for patients who received IV thrombolysis. **eFigure 2.** Distribution of mRS scores at day 90 for patients who did not receive IV thrombolysis. **eAppendix.** Detail of Additional Contributions.

Supplement 2. Protocol.

Supplement 3. Data Sharing Statement.
