# Supplementary material for: Effect of an Enhanced Paramedic Acute Stroke Treatment Assessment on Thrombolysis Delivery During Emergency Stroke Care: A Cluster Randomized Clinical Trial
Source: JAMA Neurol. 2020 Apr 13;77(7):1–9. doi: 10.1001/jamaneurol.2020.0611 (PMC7154959; doi:10.1001/jamaneurol.2020.0611)
Supplement: Supplement 1. — eTable 1. Participating hospital site characteristics. eMethods. Detailed description of the PASTA pathway. eTable 2. Permitted local variations to the PASTA pathway. eTable 3. Demography and clinical characteristics according to study group and receipt of IV thrombolysis. eTable 4. Key ambulance time intervals. eTable 5. Other acute care in hospital and key hospital time intervals. eTable 6. Full details recorded about PASTA pathway delivery. eFigure 1. Distribution of mRS scores at day 90 for patients who received IV thrombolysis. eFigure 2. Distribution of mRS scores at day 90 for patients who did not receive IV thrombolysis. eAppendix. Detail of Additional Contributions. [file jamaneurol-77-840-s001.pdf]

Supplementary Online Content

Price CI, Shaw L, Islam S, et al. Effect of an enhanced Paramedic Acute Stroke Treatment Assessment (PASTA) on thrombolysis delivery during emergency stroke care: a cluster-randomized controlled trial. *JAMA Neurol*. Published online April 13, 2020. doi:10.1001/jamaneurol.2020.0611

eTable 1. Participating hospital site characteristics

eMethods. Detailed description of the PASTA pathway

eTable 2. Permitted local variations to the PASTA pathway

eTable 3. Demography and clinical characteristics according to study group and receipt of IV thrombolysis

eTable 4. Key ambulance time intervals

eTable 5. Other acute care in hospital and key hospital time intervals

eTable 6. Full details recorded about PASTA pathway delivery

eFigure 1. Distribution of mRS scores at day 90 for patients who received IV thrombolysis

eFigure 2. Distribution of mRS scores at day 90 for patients who did not receive IV thrombolysis

eAppendix. Detail of Additional Contributions

This supplementary material has been provided by the authors to give readers additional information about their work.

© 2020 Price CI, et al. *JAMA Neurology.*

eTable 1. Participating hospital site characteristics

Ambulance service	Hospital site	Average annual stroke admissions (April 2016 - March 2018)	Average annual thrombolysis rate % (April 2016 - March 2018	Interventional neuroradiology on site	Telemedicine available for use in acute care	Number of consultants on thrombolysis rota	% stroke specialists on thrombolysis rota	National Clinical Guideline compliant for specialist thrombolysis provision
1	A	197	13	No	No	11	0	No
1	B	252	17	No	Yes	12	0	No
1	C	594	16	Yes	Yes	13	100	Yes
2	D	500	10	No	Yes	3	66	No
2	E	625	9	Yes	Yes	3	100	No
2	F	688	9	No	Yes	4	0	No
2	G	1081	10	No	Yes	7	100	Yes
2	H	1120	12	No	Yes	7	86	No
2	I	2073	9	Yes	No	13	100	Yes
3	J	608	13	No	Yes	6	66	No
3	K	656	11	No	Yes	6	100	Yes
3	L	749	12	No	Yes	7	100	Yes
3	M	817	14	No	Yes	5	80	No
3	N	911	14	Yes	No	6	100	Yes
3	O	1001	12	No	Yes	7	100	Yes

Hospital sites are listed by regional ambulance service according to increasing admission volume. Average annual stroke admissions and whole service thrombolysis rates are taken from Stroke Sentinel National Audit Programme Clinical Audit Reports (https://www.strokeaudit.org/results/Clinical-audit/National-Results.aspx). Service description data reflect hospital characteristics on 1 July 2016. These are taken from the Stroke Sentinel National Audit Programme Acute Organisational Audit Report 2016 which included all acute stroke services in England, Wales and Northern Ireland (https://www.strokeaudit.org/results/Organisational/National-Organisational.aspx). The National Clinical Guidelines for Stroke 2016 (5th Edition) recommend that thrombolysis active services should be supported by a continuous specialist rota comprising a minimum of six physicians with training in emergency stroke assessment (https://www.strokeaudit.org/SupportFiles/Documents/Guidelines/2016-National-Clinical-Guideline-for-Stroke-5t-(1).aspx).

© 2020 Price CI, et al. *JAMA Neurology.*

eMethods. Detailed description of the PASTA pathway

PASTA trained paramedics were asked to deliver the PASTA pathway to the following patients: aged 18 years and over; Face Arm Speech Test (FAST) positive or any presentation of new focal neurological symptoms which indicated acute stroke in the paramedic's routine clinical judgement; within 4 hours of last known to be without new stroke symptoms in the paramedic’s judgement; admission to a study hospital.

The pathway consisted of the following stages:

1. Information. The paramedic sought additional information at the scene which is routinely considered during IV thrombolysis treatment decisions but typically is not obtained until after hospital admission. This included the presence of additional stroke symptoms (dysphasia and visuospatial deficits), prescription of anticoagulant medication, a recent medical history of surgery or bleeding, any previous history of transient ischaemic attack (TIA) or stroke and the current level of dependency (direct assistance required for feeding or walking). These information categories were summarised by the acronym PASTA which represented: Plus dysphasia or visuospatial impairment; Anticoagulant medications; Surgery or other bleeding recently; TIA or stroke previously; Assistance needed daily.

2. Pre-notification. Although an expected component of SC, PASTA paramedics were specifically asked (reminded) to warn the destination hospital in accordance with existing local arrangements.

3. Handover. On arrival the paramedic provided a standardised handover of stroke-specific details to the hospital team including FAST, onset time, patient alertness using the AVPU scale [McNarry AF, Bateman DN. Simple bedside assessment of level of consciousness: comparison of two simple assessment scales with the Glasgow Coma scale. Anaesthesia 2004; 59(1): 34–7] and PASTA information. . They indicated any information which was unavailable at the scene and reminded the receiving hospital team about optimal time targets for scan (< 15 min) and IV thrombolysis treatment (< 30 min).

4. Scan. If the CT scan was immediately available, the paramedic assisted with patient transfer to the scan room.

5. Assist. Up until 15 minutes after arrival, the paramedic undertook the following tasks as required: insertion of an intravenous cannula; obtaining the patient’s weight; and repetition/clarification of clinical information for arriving stroke team members.

6. Checklist. At 15 min after handover the paramedic asked a member of the hospital team to confirm progress with key tasks: the status of the scan request and stroke specialist review (if not yet performed); confirmation of relevant medical history; and blood tests for clotting indices if required.

7. Feedback. The paramedic requested feedback from a hospital clinician about the accuracy of their provisional stroke diagnosis and onset time estimation.

Local variations to the pathway were permitted as shown in Supplementary eTable 2.

© 2020 Price CI, et al. *JAMA Neurology.*

eTable 2. Permitted local variations to the PASTA pathway

PASTA component	Core content expected to be delivered	Local variation permitted
Information	Must use PASTA format.	Any clinical information collection system (paper/electronic)
Pre-notification	Must be performed for all PASTA admissions.	Paramedic call to ED.<br>Paramedic call to HASU.<br>Dispatch call to ED.<br>Dispatch call to HASU.<br>Pre-notification may or may not include patient identifiable information.
Handover	Must follow study format.	Occurs in most suitable area for rapid handover and registration.<br>Receiving hospital team are ED and/or stroke service clinicians.<br>May include other relevant information for individual patients.
Scan	Paramedic assists with transfer to scan if <15minutes since handover.	Any appropriate hospital clinician accompanies patient.<br>Any brain imaging modality which would assist treatment decisions.<br>Transfer by ambulance or hospital trolley as long as no delay incurred.
Assist	Patient transferred off ambulance trolley onto hospital trolley.<br>Paramedic offers assistance for initial tasks.<br>Ambulance crew prepares for departure.	Team may not require assistance for each or all of tasks.<br>Ambulance operational conditions may shorten paramedic stay.
Checklist	Paramedic enquires about progress from any hospital team member at 15 minutes since handover.	Checklist completion should reflect usual local service procedures (e.g. telemedicine specialist review)
Feedback	Paramedic seeks feedback about provisional diagnosis and onset time.	Any member of hospital team can provide feedback.<br>Paramedic can seek feedback on any aspect of the assessment.<br>The hospital team may not (yet) be able to provide feedback.

© 2020 Price CI, et al. *JAMA Neurology.*

eTable 3. Demography and clinical characteristics according to study group and receipt of IV thrombolysis

	PASTA – thrombolysed	PASTA – not thrombolysed	Standard care – thrombolysed	Standard care – not thrombolysed
**Gender n (%)**	**n=197**	**n=303**	**n=319**	**n=395**
Male	110 (55·8)	149 (49·2)	167 (52·4)	198 (50·1)
Female	87 (44·2)	154 (50·8)	152 (47·6)	197 (49·9)
**Age (years)**	**n=197**	**n=303**	**n=319**	**n=395**
Median [IQR]	75 (63-82)	78 (70- 85)	75 (64-82)	79 (70-85)
**PRE-STROKE Modified Rankin Scale Score n(%)**	**n=195**	**n=299**	**n=318**	**n=390**
0	117 (60·0)	116 (38·8)	184 (57·9)	157 (40·3)
1	26 (13·3)	52 (17·4)	55 (17·3)	71 (18·2)
2	24 (12·3)	41 (13·7)	35 (11·0)	44 (11·3)
3	18 (9·2)	48 (16·1)	31 (9·7)	66 (16·9)
4	10 (5·1)	32 (10·7)	12 (3·8)	35 (9·0)
5	0	10 (3·3)	1 (0·3)	17 (4·4)
0-2 n(%)	167 (85·6)	209 (69·9)	274 (86·2)	272 (69·7)
3-5 n(%)	28 (14·4)	90 (30·1)	44 (13·8)	118 (30·3)
**Stroke severity at admission (NIHSS)**	**n=197**	**n=302**	**n=318**	**n=392**
Median [IQR]	11 (6-19)	7 (3-17)	11 (6-18)	7 (3-19)
Mean [SD]	12·3 (7·5)	10·4 (9·4)	12·4 (7·4)	10·8 (9·3)
**Results of the first brain imaging n(%)**	**n=197**	**n=302**	**n=319**	**n=395**
Infarction	196[a] (99·5)	213 (70·5)	319 (100)	288 (72·9)
Primary intracerebral haemorrhage (ICH)	1 (0·5)	89 (29·5)	0	106 (26·8)
Other	0	0	0	1 (0·3)
**Blood pressure on admission-systolic**	**n=196**	**n=301**	**n=317**	**n=395**
Median [IQR]	152 (132-169)	162 (140-185)	151 (136-168)	158 (138-182)
Mean [SD]	151·0 (25·4)	162 (33)	152·7 (25·9)	161·0 (32·0)
**Blood pressure on admission-diastolic**	**n=196**	**n=301**	**n=317**	**n=395**
Median [IQR]	82 (70-94)	86 (74-99)	80 (71-89)	85 (72-96)
Mean [SD]	82·4 (17·4)	86·9 (19·8)	81·2 (16·6)	85·7 (19·1)
**Blood glucose on admission**	**n=190**	**n=288**	**n=303**	**n=379**
Median [IQR]	6·7 (5·7–7·8)	6·7 (5·7-7·9)	6·5 (5·7-7·8)	6·7 (5·7-8·4)

© 2020 Price CI, et al. *JAMA Neurology.*

	PASTA – thrombolysed	PASTA – not thrombolysed	Standard care – thrombolysed	Standard care – not thrombolysed
Mean [SD]	7·2 (2·6)	7·5 (2·9)	7·1 (2·3)	7·7 (3·8)
**Anticoagulation on admission n(%)**	**n=197**	**n=302**	**n=319**	**n=394**
Warfarin	6 (3·0)	36 (11·9)	11 (3·4)	40 (10·1)
Apixaban	0	9 (3·0)	0	24 (6·1)
Rivaroxaban	1 (0·5)	11 (3·6)	3 (0·9)	19 (4·8)
Dabigatran	1 (0·5)	2 (0·7)	1 (0·3)	1 (0·3)
**Location of first hospital assessment n(%)**	**n=197**	**n=303**	**n=319**	**n=395**
Accident and Emergency Department	135 (68·5)	213 (70·3)	213 (66·8)	312 (79·0)
CT scan room	9 (4·6)	15 (5·0)	16 (5·0)	10 (2·5)
Acute Stroke Unit	43 (21·8)	53 (17·5)	82 (25·7)	55 (13·9)
Critical care (ITU, HDU, CCU)	4 (2·0)	0	0	1 (0·3)
Medical admissions unit	6 (3·0)	21 (6·9)	8 (2·5)	17 (4·3)
Unknown	0	0	0	0
Other	0	1 (0·3)	0	0

[a] This value is 196 not 197 because one patient with subtle haemorrhagic stroke not initially identified on the admission CT received thrombolysis

© 2020 Price CI, et al. *JAMA Neurology.*

eTable 4. Key ambulance time intervals

Time interval (minutes)	PASTA	Standard care	Difference in mean (PASTA-SC) (95% CI)	p value
**Stroke onset to 999 call**	**n=454**	**n=623**	-3·35 (-9·50 to 2·80)	0·28
Mean [SD]	47·4 (51·7)	50·8 (49·7)		
Median [IQR]	26 (9-67·0)	32 (12-76·0)		
**999 call to paramedic assessment**	**n=479**	**n=681**	0·75 (-1·98 to 3·50)	0·59
Mean [SD]	28·5 (23·3)	27·7 (23·3)		
Median [IQR]	22 (14-36)	20 (14-34)		
**Paramedic assessment to leave scene**	**n=451**	**n=615**	1·61 (-0·20 to 3·42)	0·08
Mean [SD]	26·0 (15·5)	24·4 (14·4)		
Median [IQR]	24 (15-34)	22 (14-31)		
**Leave scene to hospital admission**	**n=441**	**n=626**	0·08 (-1·14 to 1·30)	0·90
Mean [SD]	16·4 (9·2)	16·3 (10·6)		
Median [IQR]	14 (10-20)	14 (9-20)		
**Hospital admission to paramedic clear**	**n=445**	**n=616**	8·80 (6·50 to 11·04)	<0·001
Mean [SD]	39·9 (20·2)	31·1 (15·6)		
Median [IQR]	36 (27-50)	29 (20-38)		
**Total 999 to paramedic clear**	**n=474**	**n=666**	13·43 (9·42 to 17·44)	<0·001
Mean [SD]	108·8 (36·3)	95·3 (30·5)		
Median [IQR]	102 (85-123)	90 (76-110)		

© 2020 Price CI, et al. *JAMA Neurology.*

eTable 5. Other acute care in hospital and key hospital time intervals

	PASTA	Standard care	Comparison
**Acute care**			**Odds ratio (95% CI, p value)**
Referral for intra-arterial treatment n(%)	**n=499**<br>13 (2·6%)	**n=714**<br>18 (2·5%)	1·03 (0·50 to 2·13, p=0·93)
Transfer for intra-arterial treatment n(%)	**n=498**<br>12 (2·4%)	**n=713**<br>15 (2·1%)	1·15 (0·53 to 2·5, p=0·72)
Referral for neurosurgical assessment n(%)	**n=499**<br>45 (9·0%)	**n=714**<br>46 (6·4%)	1·4 (0·94 to 2·21, p=0·09)
Transfer for neurosurgical assessment n(%)	**n=499**<br>4 (0·8%)	**n=713**<br>9 (1·3%)	0·63 (0·19 to 2·1, p=0·44)
Intravenous blood pressure lowering pre-thrombolysis n(%)	**n=197**<br>27 (13·7%)	**n=319**<br>39 (12·2%)	1·14 (0·67 to 1·93, p=0·63)
Intravenous blood pressure control in haemorrhagic stroke n(%)	**n=90**<br>39 (43·3%)	**n=106**<br>47 (44·3%)	0·96 (0·55 to 1·79, p=0·89)
Reversal of abnormal coagulation for haemorrhagic stroke n(%)	**n=90**<br>13 (14·4%)	**n=106**<br>16 (15·1%)	0·95 (0·43 to 2·1, p=0·90)
Received one or more of the above interventions n(%)	**n=500**<br>133 (22·6%)	**n=714**<br>143 (20·0%)	1·16 (0·88 to 1·54, p=0·28)
**Time intervals**			**Difference in mean PASTA-SC (95% CI, p value)**
**Paramedic assessment to brain imaging time (mins)**	**n=490**	**n=700**	3·60 (-9·5 to 16·71, p=0·59)
Mean [SD]	88.9 (127.7)	85.4 (102.4)	
Median [IQR]	65 (50-89)	66 (50-90)	
**Hospital arrival to brain imaging time (mins)**	**n=450**	**n=648**	-0·61 (-14·05 to 12·84, p=0·93)
Mean [SD]	47.0 (123.8)	47.6 (102.4)	
Median [IQR]	23 (16-40)	26 (15-45)	

© 2020 Price CI, et al. *JAMA Neurology.*

eTable 6. Full details recorded about PASTA pathway delivery

Forms received	n=227
**PASTA information 1: speech and vision: n (%)**	
**How is speech:**	
Normal	31 (13·7)
Slurred	79 (34·8)
With word finding difficulties	41 (18·1)
Slurred with word finding difficulties	63 (27·8)
Missing	*13 (5·7)*
**Is the speech issue new:**	
Yes	10 (4·4)
No	171 (75·3)
Unknown	8 (3·5)
Missing	*38 (16·7)*
**Known to have visual problems:**	
Yes	44 (19·4)
No	133 (58·6)
Unknown	36 (15·9)
Missing	*14 (6·2)*
**How is vision:**	
Patient can see both left and right sides	104 (45·8)
Patient can see left side only	10 (4·4)
Patient can see right side only	18 (7·9)
Patient unable to see both left and right sides	4 (1·8)
Patient unable to understand instructions	65 (28·6)
Missing	*26 (11·5)*
**PASTA information 2: anticoagulants: n (%)**	
**Warfarin**	
Yes	23 (10·1)
No	179 (78·9)
Unknown	9 (4·0)
Missing	*16 (7·0)*
**Apixaban**	
Yes	4 (1·8)
No	195 (85·9)
Unknown	10 (4·4)
Missing	*18 (7·9)*
**Rivaroxaban**	
Yes	5 (2·2)
No	192 (84·6)
Unknown	11 (4·8)
Missing	*19 (8·4)*

© 2020 Price CI, et al. *JAMA Neurology.*

Forms received	n=227
**Dabigatran**	
Yes	2 (0·9)
No	195 (85·9)
Unknown	11 (4·8)
Missing	*19 (8·4)*
**Takes unknown anticoagulant**	
Yes	15 (6·6)
No	117 (51·5)
Unknown	0
Missing	*95 (41·9)*
**PASTA information 3: surgery: n (%)**	
**Had surgery or bleeding in last 3 months?**	
Yes	18 (7·9)
No	186 (81·9)
Unknown	8 (3·5)
Missing	*15 (6·6)*
**If yes, number of weeks ago:**	
Min	0
Max	17
Median	4
IQR (Q1, Q3)	6 (2,8)
Mean	5·1
SD	4·2
Missing	*1 (5·6)*
**PASTA information 4: previous TIA/stroke: n (%)**	
**Previous TIA:**	
Yes	49 (21·6)
No	152 (67·0)
Unknown	13 (5·7)
Missing	*13 (5·7)*
**Previous Stroke:**	
Yes	37 (16·3)
No	167 (73·6)
Unknown	10 (4·4)
Missing	*13 (5·7)*
**Previous haemorrhage:**	
Yes	6 (2·6)
No	185 (81·5)
Unknown	11 (4·8)
Missing	*25 (11·0)*
**PASTA information 5: assistance: n (%)**	
**Walking:**	

© 2020 Price CI, et al. *JAMA Neurology.*

**Forms received**	**n=227**
Yes	26 (11·5)
No	196 (86·3)
Unknown	2 (0·9)
Missing	*3 (1·3)*
**Eating:**	
Yes	8 (3·5)
No	211 (93·0)
Unknown	5 (2·2)
Missing	3 (1·3)
**Pre-notification: n (%)**	
**A+E:**	
Not attempted	84 (37·0)
Unsuccessful	0
Successful	81 (35·7)
Missing	*62 (27·3)*
**Stroke team:**	
Not attempted	92 (40·5)
Unsuccessful	4 (1·8)
Successful	61 (26·9)
Missing	*70 (30·8)*
**Via dispatch/control:**	
Not attempted	41 (18·1)
Unsuccessful	0
Successful	129 (56·8)
Missing	*57 (25·1)*
**Total successful pre-notification recorded**	219 (96·5)
**Pathway deviation: n (%)**	
Yes	2 (0·9)
No	214 (94·3)
Missing	*11 (4·8)*
**Handover: n (%)**	
**Did the handover use FASTA PASTA CT format?**	
Yes	134 (59·0)
No	8 (3·5)
Missing	*85 (37·4)*
**Paramedic actions: n (%)**	
**Transfer directly to scan**	
Yes	43 (18·9)
No	160 (70·5)
Missing	*24 (10·6)*
**Transfer to scan from A+E/stroke unit**	
Yes	101 (44·5)

© 2020 Price CI, et al. *JAMA Neurology.*

Forms received	n=227
No	73 (32·2)
Missing	*53 (23·3)*
**IV cannula before admission**	
Yes	124 (54·6)
No	97 (42·7)
Missing	*6 (2·6)*
**IV cannula after admission**	
Yes	53 (23·3)
No	114 (50·2)
Missing	*60 (26·4)*
**Weight measurement/estimation**	
Yes	69 (30·4)
No	121 (53·3)
Missing	*37 (16·3)*
**Clarification/repetition of handover**	
Yes	190 (83·7)
No	15 (6·6)
Missing	*22 (9·7)*
**Checklist: n (%)**	
**Brain scan requested**	
Yes	184 (81·1)
No	14 (6·2)
Missing	*29 (12·8)*
**Stroke team aware**	
Yes	192 (84·6)
No	9 (4·0)
Missing	*26 (11·5)*
**Stroke team reviewed**	
Yes	167 (73·6)
No	27 (11·9)
Missing	*33 (14·5)*
**Patient medical history known**	
Yes	201 (88·5)
No	4 (1·8)
Missing	*22 (9·7)*
**Patient medication known**	
Yes	190 (83·7)
No	14 (6·2)
Missing	*23 (10·1)*
**Blood clotting test requested**	
Not relevant	40 (17·6)
Yes	73 (32·2)

© 2020 Price CI, et al. *JAMA Neurology.*

Forms received	n=227
No	17 (7·5)
Missing	*97 (42·7)*
**Feedback**	
**Feedback received?**	
Yes	156 (68·7)
No	44 (19·4)
Missing	*27 (11·9)*
If yes:	
**Diagnosis feedback**	
Yes	138 (88·5)
No	14 (9·0)
Missing	*4 (2·6)*
**Onset time feedback**	
Yes	113 (72·4)
No	24 (15·4)
Missing	*19 (12·2)*
**Other feedback**	
Yes	43 (27·6)
No	56 (35·9)
Missing	*57 (36·5)*
**Did the paramedic leave the patient early (ie <15mins since handover): n (%)**	
Yes: assistance no longer needed	43 (18·9)
Yes: at control request	4 (1·8)
No	166 (73·1)
Missing	*14 (6·2)*

© 2020 Price CI, et al. *JAMA Neurology.*

eFigure 1. Distribution of mRS scores at day 90 for patients who received IV thrombolysis

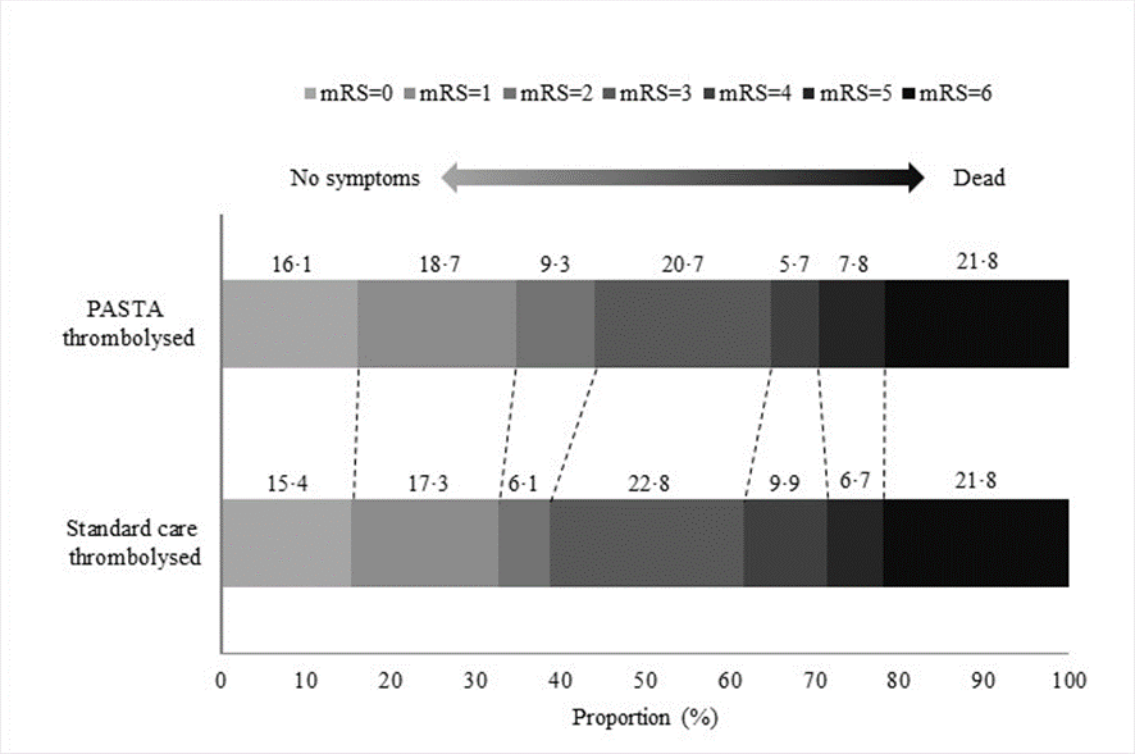

© 2020 Price CI, et al. *JAMA Neurology.*

eFigure 2. Distribution of mRS scores at day 90 for patients who did not receive IV thrombolysis

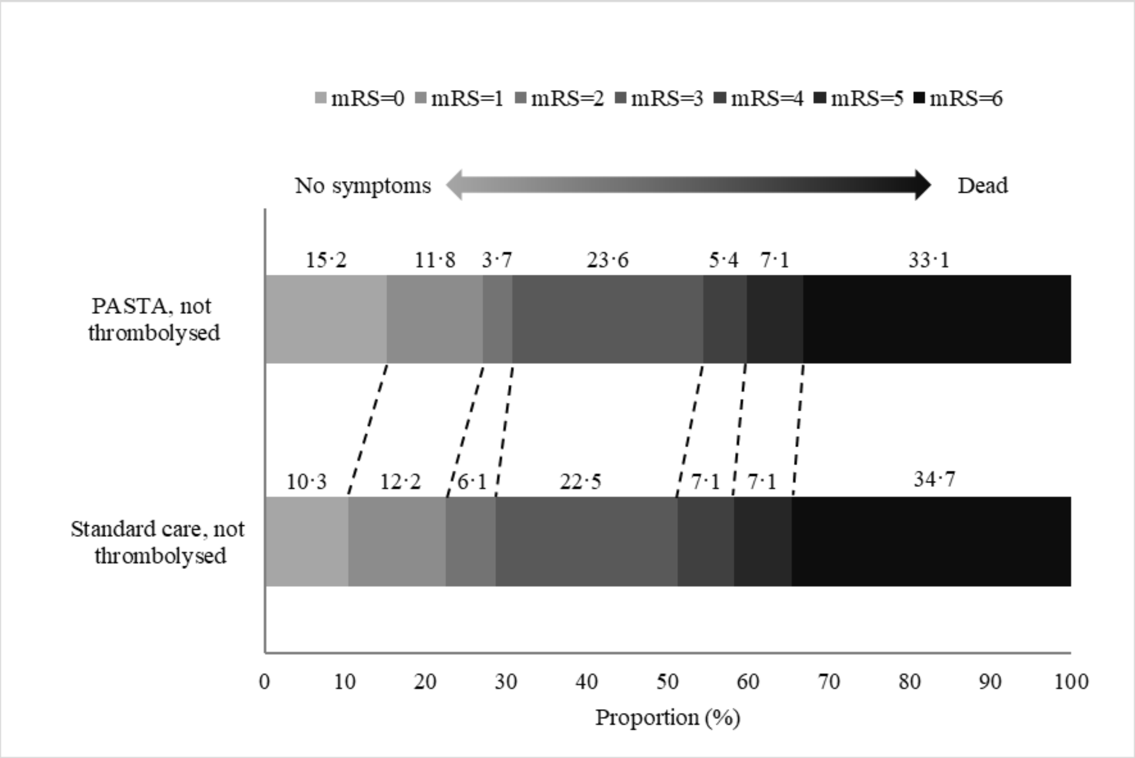

© 2020 Price CI, et al. *JAMA Neurology.*

eAppendix. Details of Additional Contributions

We acknowledge the support of the following research teams in ambulance services and hospitals: North East Ambulance Service NHS Trust: Mr Paul Aitken-Fell; Newcastle Hospitals NHS Foundation Trust (Royal Victoria Infirmary): Dr Omar (Mustafa) Hossain; Northumbria Healthcare NHS Foundation Trust (Northumbria Specialist Emergency Care Hospital): Dr Chris Price; County Durham and Darlington NHS Foundation Trust (University Hospital North Durham): Sister Michelle Matthews; South Tyneside and Sunderland NHS Foundation Trust (Sunderland Royal Hospital): Dr Naweed Sattar; North Tees and Hartlepool NHS Foundation Trust (University Hospital of North Tees): Dr Arun Annamalai; South Tees Hospitals NHS Foundation Trust (James Cook University Hospital): Dr Adrian Bergin; North West Ambulance Service NHS Trust: Mr Duncan Robertson/Mrs Caitlin Wilson; Pennine Acute Hospitals NHS Trust (Fairfield General Hospital): Dr Khalil Kawafi; Stockport NHS Foundation Trust (Stepping Hill Hospital): Dr Joseph Vassallo; Salford Royal NHS Foundation Trust (Salford Royal Hospital): Ms Sarah Heathcote; East Lancashire Hospitals NHS Trust (Royal Blackburn Hospital): Catherine Curley; Blackpool Teaching Hospitals NHS Foundation Trust (Blackpool Victoria Hospital): Alex Maley; Lancashire Teaching Hospitals NHS Foundation Trust (Royal Preston Hospital): Prof Hedley Emsley; Welsh Ambulance Services NHS Trust: Mr Chris Moore; Cardiff and Vale University Health Board (University Hospital of Wales): Dr Hamsaraj Shetty; Hywel Dda University Health Board (Prince Philip Hospital and Glangwili General Hospital): Dr Sentil Kumar Subbarayan.

We also thank the external Data Monitoring Committee members (Tom Robinson (chair), Caroline Watkins, Tom Quinn, Chris Sutton), Trial Steering Committee members (Charles Wolfe (chair), Martin James, Niro Siriwardena, Amanda Farrin, Olivia Wu, Christine Roffe, Damian Jenkinson, Michael Speed), co-investigator Pippa Tyrell who retired during the study and patient/public representatives of the project team (Peter Dodd, Bill Laing, Melissa Roberts).

© 2020 Price CI, et al. *JAMA Neurology.*
