# Supplementary material for: Effect of an Enhanced Paramedic Acute Stroke Treatment Assessment on Thrombolysis Delivery During Emergency Stroke Care: A Cluster Randomized Clinical Trial
Source: JAMA Neurol. 2020 Apr 13;77(7):1–9. doi: 10.1001/jamaneurol.2020.0611 (PMC7154959; doi:10.1001/jamaneurol.2020.0611)
Supplement: Supplement 2. — Protocol. [file jamaneurol-77-840-s002.pdf]

Paramedic Acute Stroke Treatment Assessment

(PASTA)

Study Protocol

Version 2: 31 October 2017

Study Funder:
Programme Grants for Applied Research
National Institute for Health Research
Central Commissioning Facility
Grange House
15 Church Street
Twickenham
TW1 3NL

Study Sponsor:
Newcastle upon Tyne Hospitals NHS Foundation Trust
Newcastle Joint Research Office
1st Floor
Regent Point
Regent Farm Road
Newcastle upon Tyne
NE3 3HD

Chief Investigator:
Dr Christopher Price
Stroke Research Group
Institute of Neuroscience
Newcastle University
3-4 Claremont Terrace
Newcastle upon Tyne
NE2 4AE

Contents

Protocol contacts 3
NIHR programme grant (objective 1) award holders 4
1. Protocol signature page 5
2. Glossary 6
3. Responsibilities 7
4. Protocol summary 8
5. Background 9
6. Study aims and objectives 10
7. Study design 11
8. Study setting 11
9. Randomisation 11
10. Study treatments 12
11. Study participants 16
12. Participant identification, recruitment and consent 17
13. Study data collection 23
14. Blinding 25
15. Staff training and awareness 25
16. Study withdrawal 26
17. Safety evaluation 26
18. Statistical analysis 28
19. Internal pilot study 30
20. Economic analysis 30
21. Qualitative evaluation 32
22. Ethics and regulatory issues 35
23. Confidentiality 35
24. Trial monitoring, quality control and quality assurance 35
25. Indemnity 36
26. Funding 36
27. Dissemination of results 36
28. References 37
Appendices 39

Protocol contacts

Chief Investigator
Name: Dr Christopher Price
Consultant Stroke Physician and Honorary Clinical Senior Lecturer
Address: Stroke Research Group
Newcastle University
3 – 4 Claremont Terrace
Newcastle upon Tyne
NE2 4AE
Phone: 0191 208 3842
Email: c.i.m.price@newcastle.ac.uk

Contact for study sponsor
Name: Susan Ridge
Research Governance Manager
Address: Newcastle upon Tyne Hospitals NHS Foundation Trust
Newcastle Joint Research Office
1st Floor
Regent Point
Regent Farm Road
Newcastle upon Tyne
NE3 3HD
Phone: 0191 282 4823
Email: susan.ridge@nuth.nhs.uk

Contact for trial management
Name: PASTA trial team
Address: Stroke Research Group
Newcastle University
3 – 4 Claremont Terrace
Newcastle upon Tyne
NE2 4AE
Phone: 0191 208 8209
Email: pasta@ncl.ac.uk

NIHR programme grant (objective 1) award holders

Lead applicant:

Professor Gary Ford, Chief Executive Officer, Oxford Academic Health Science Network, John Eccles House, Robert Robinson Avenue, Oxford Science Business Park OX4 4GP. Email: gary.ford@ouh.nhs.uk

Co-applicants:

Mr Peter Dodd, Lay member, Contact via: Stroke Research Group, Institute of Neuroscience, Newcastle University, 3-4 Claremont Terrace, Newcastle upon Tyne, NE4 4AE.

Professor Catherine Exley, Faculty Associate Pro Vice-Chancellor, Research and Innovation, Faculty of Health & Life Sciences, Northumbria University, 2nd floor Northumberland Building, Newcastle upon Tyne, NE1 8ST. Email: catherine.exley@northumbria.ac.uk.

Mr Paul Fell, Acting Director of Clinical Care and Patient Safety, North East Ambulance Service, Bernicia House, Goldcrest Way, Newburn Riverside, Newcastle upon Tyne, NE15 8NY. Email: paul.fell@neas.nhs.uk

Dr Darren Flynn, Senior Research Associate, Institute of Health and Society, Newcastle University, Baddiley-Clark Building, Richardson Road, Newcastle upon Tyne, NE2 4AX. Email: darren.flynn@newcastle.ac.uk

Dr Peter McMeekin, Faculty of Health and Life Sciences, Northumbria University, Coach Lane Campus, Newcastle upon Tyne, NE7 7XA. Email: peter.mcmeekin@northumbria.ac.uk

Dr Christopher Price, Clinical Senior Lecturer in Medicine, Stroke Research Group , Institute of Neuroscience, Newcastle University, 3-4 Claremont Terrace, Newcastle upon Tyne, NE2 4AE. Email: c.i.m.price@newcastle.ac.uk

Professor Helen Rodgers, Professor of Stroke Care, Stroke Research Group, Institute of Neuroscience, Newcastle University, 3-4 Claremont Terrace, Newcastle upon Tyne, NE2 4AE. Email: helen.rodgers@ncl.ac.uk

Professor Ian Russell, Professor of Clinical Trials, College of Medicine, Swansea University, Singleton Park, Swansea, SA2 8PP, Wales. Email: i.t.russell@swansea.ac.uk

Professor Helen Snooks, Professor of Health Services Research, College of Medicine, Swansea University, Singleton Park, Swansea, SA2 8PP, Wales. Email: h.a.snooks@swansea.ac.uk

Professor Pippa Tyrrell, Professor of Stroke Medicine, Stroke Medicine, Clinical Sciences Building, Salford Royal Hospitals' Foundation Trust, Salford, M6 8HD. Email: pippa.tyrrell@manchester.ac.uk

Professor Luke Vale, Health Foundation Chair in Health Economics, Institute of Health and Society, Newcastle University, Baddiley-Clark Building, Richardson Road, Newcastle upon Tyne, NE2 4AX. Email: luke.vale@newcastle.ac.uk

1. Protocol signature page

Local site principal investigator signature:

I have read and agree to version 2 of the protocol, dated 31 October 2017 entitled "**Paramedic Acute Stroke Treatment Assessment (PASTA)**".

I am aware of my responsibilities as an Investigator under the Research Governance Framework for Health and Social Care. I agree to conduct the study according to these guidelines and to appropriately direct and assist the staff under my control, who will be involved in the study.

Name…………………………Signature ……………………………… Date …………

Affiliation……………………..

2. Glossary

Abbreviation	Definition
AE	Adverse Event
AVPU	Alert Voice Pain Unresponsive
CRF	Case Record Form
CT	Computerised Tomography
CTNT	Call To Needle Time
CTU	Clinical Trial Unit
DMEC	Data Monitoring and Ethics Committee
DTNT	Door To Needle Time
DTST	Door To Scan Time
ED	Emergency Department
FAST	Face Arm Speech Time
iv rtPA	Intravenous recombinant tissue plasminogen activator
mRS	Modified Rankin Scale
NIHR	National Institute for Health Research
NIHSS	National Institute of Health Stroke Scale
NHS	National Health Service
NRES	National Research Ethics Service
CTNT	Call To Needle Time
CTST	Call To Scan Time
PI	Principal Investigator
PIS	Patient Information Sheet
QALY	Quality-Adjusted Life-Year
RCT	Randomised Controlled Trial
R+D	Research and Development
REC	Research Ethics Committee
SAE	Serious Adverse Event
SITS	Safe Implementation of treatments in Stroke
TMG	Trial Management Group
TSC	Trial Steering Committee

3. Responsibilities

Funder: The National Institute for Health Research is funding this study.
Sponsor: Newcastle upon Tyne Hospitals NHS Foundation Trust is the sponsor for this study.

Overall Trial Management:

The following functions falling under the responsibility of the sponsor are delegated to the Chief Investigator, Dr Christopher Price:

- Ethics Committee Opinion (including application for research ethics committee favourable opinion, notification of protocol amendments and end of trial)
- R&D Approval (including application for global checks, via NIHR CSP)
- Good Clinical Practice and Trial Conduct (including GCP arrangements, data monitoring, emergency & safety procedures)
- Administration of funding for the study

A Trial Management Group (TMG) will be appointed and will be responsible for overseeing the progress of the trial. The day-to-day management of the trial will be co-ordinated by Newcastle Clinical Trials Unit and the Newcastle University Stroke Research Group.

Trial conduct at site:

A Principal Investigator will have overall responsibility for the conduct of the study at a trial site.

Investigator responsibilities:

- Study conduct and the welfare of study participants
- Familiarity with the study intervention(s).
- Compliance with the protocol, documentation of any protocol deviations and reporting of all serious adverse events
- Identification of participants
- Ensuring all trial-related medical decisions are made by a qualified physician, who is an investigator or co-investigator for the trial.
- Provision of adequate medical care in the event of an adverse event
- Obtaining local approval and abiding by the policies of Research Governance
- Compliance with the Principles of GCP, the Research Governance Framework for Health and Social Care, the Data Protection Act and any other relevant legislation and regulatory guidance.
- Ensuring that no participant is recruited into the study until all relevant regulatory permissions and approvals have been obtained.
- The Principal Investigator (PI) shall be qualified by education, training and experience to assume responsibility for the proper conduct of the trial. S/he shall provide a current signed & dated curriculum vitae as evidence for the Trial Master File.
- Ensuring study site team members are appropriately qualified by education, training and experience to undertake the conduct of the study.
- Availability for any site meetings, monitoring visits and in the case of an audit.
- Maintaining study documentation and compliance with reporting requests
- Maintaining a site file, including copies of study approval, list of participants and their signed informed consent forms
- Documenting appropriate delegation of tasks to other study personnel e.g. Research Nurse, Co-Investigator(s), Trial Coordinators, Data Managers
- Ensuring data collected is accurate, timely & complete
- Providing updates on the progress of the trial
- Ensuring participant confidentiality is maintained during the project and archival period
- Ensuring archival of study documentation for a minimum of 5 years following the end of the study, unless local arrangements require a longer period

4. Protocol summary

**Title:**	Paramedic Acute Stroke Treatment Assessment (PASTA)
**Chief Investigator:**	Dr Christopher Price
**Sponsor**:	Newcastle upon Tyne Hospitals NHS Foundation Trust
**Funder:**	National Institute for Health Research Programme Grants for Applied Research Programme (RP-PG-1211-20012).
**Study design**:	A cluster randomised trial with cost-effectiveness analysis and parallel process evaluation.
**Study setting**:	NHS ambulance services, emergency departments and stroke units within England and Wales.
**Study intervention:**	A Paramedic Acute Stroke Treatment Assessment (PASTA) pathway initiated by paramedics and continued initially in hospital to facilitate the speed of brain imaging and delivery of thrombolysis when clinically appropriate.
**Study control**:	Usual care according to national and local guidelines for the pre-hospital and hospital assessment of suspected stroke.
**Randomisation**:	Ambulance stations or paramedic teams (according to local service configuration) within each region randomised to delivering the PASTA pathway or to continue with usual stroke care.
**Study participants**	Adults with confirmed stroke who were assessed by a study paramedic within 4 hours of onset and transported to a study hospital.
**Primary outcome**:	Proportion of participants receiving intravenous thrombolysis.
**Secondary outcomes**:	• Stroke severity and complications at 24 hrs post thrombolysis<br>• Inpatient mortality<br>• Destination at discharge and 90 days<br>• Dependency at discharge and 90 days<br>• Assistance required at discharge and 90 days<br>• Time from emergency call and hospital admission to first brain imaging, thrombolysis, stroke unit admission, formal swallow assessment<br>• Patient experiences during the intervention pathway<br>• Professional views of the intervention pathway
**Sample size**:	1297 participants provide 90% power to detect a 10% difference in the proportion of patients receiving thrombolysis.
**Study duration**:	2.5 Years.

5. Background

Stroke is responsible for a high global burden of mortality and disability[1]. In the UK it remains the third leading cause of death and the single largest cause of adult disability with an economic impact of approximately £4 billion per year[2].

The most widely used cost-effective emergency treatment is thrombolysis using intravenous recombinant tissue Plasminogen Activator (iv rtPA) for selected ischaemic stroke cases within 4.5 hours of symptom onset, but outcomes are highly time dependent[3] [4]. Within 60-90 minutes of symptom onset only 4 suitable patients need to be treated for one person to be free from disability, whereas at 270 minutes the same impact would require 9 patients to be treated. Despite the evidence from clinical trials, related guidelines and policy, audit continues to show large variations in the rate and speed of thrombolysis delivery between services and diurnal variations within services[5, 6]. In 2014, only 11% of total stroke admissions in the NHS were treated with thrombolysis against an aspirational target of 20%, with a median door to treatment time of 54 minutes despite a target of <40 minutes[5]. Benefits for patients and social care resources would be substantially improved if more patients were treated sooner.

Most stroke services have found considerable challenges in reducing iv rtPA treatment delays in hospital (i.e. door to needle time: DTNT), particularly, rapid access to brain imaging (i.e. door to scan time: DTST). Brain imaging is a vital component of assessment to exclude haemorrhagic stroke and patients with established ischaemic changes where thrombolysis would be futile. International clinical guidelines state that brain imaging should be performed immediately when a patient with thrombolysis potential arrives[7, 8]. An urgent scan is also indicated for other patients presenting with stroke symptoms including those taking anticoagulation medication and when a haemorrhage is suspected. Urgent treatments for these patients can include reversal of anticoagulation, intravenous medication to lower high blood pressure and possible neurosurgical intervention. Although across the NHS brain imaging was achieved within 1 hour for 43% of stroke admissions in 2014 there was wide variation between services and the low thrombolysis rate suggests that patients with the most to gain from rapid radiological assessment were not actively being selected[5]. An improvement in the early identification of patients who meet the criteria for an urgent scan could lead to a cost-effective reduction in dependency, mainly through an increase in the speed of iv rtPA treatment but also by improving access to other treatments and organised stroke care.

To date, most service interventions to reduce DTST and DTNT have focussed upon responses after patient admission to the Emergency Department (ED) or Acute Stroke Unit (ASU). Where improvements have been seen this usually reflects highly resourced centres, but even these may rely upon initial patient assessment by nursing and junior medical staff at nights and weekends. In some settings, the standard approach is remote stroke specialist assessment by video-link or telephone, increasing the reliance upon non-specialist staff for rapid and accurate information collection and communication at the bedside.

In the pre-hospital setting, there is good evidence that the paramedic sensitivity for stroke identification using the Face Arm Speech Test (FAST) is equivalent to that of non-specialist ED staff [9] and that ambulance contact to the ED to provide advance notice of admission (pre-notification) can have a positive impact [10, 11]. However despite existing operational guidelines within ambulance services to encourange identification of suspected stroke and symptom onset time, pre-notification does not occur systematically in clinical practice and the significance of other information collected by paramedics may not be realised during patient handover at hospital (e.g. current medication and recent medical history). Although healthcare policy supports ongoing development of the paramedic role[12], there has been no rigorous examination of how paramedics could best contribute to improving DTNT and DTST. A recent Swedish randomised trial showed that thrombolysis rates and hospital treatment delays significantly improved after providing paramedics with training and raising the emergency status of stroke ambulance dispatch[13]. An alternative model of “mobile stroke units”

(adapted ambulances with CT scanner and neurologist on board or video link to a stroke specialist) has been shown to reduce service-level call to needle times (CTNT) by 15 minutes in dense urban areas, but with no change in outcomes, additional costs and uncertain technological reliability[14-16]. As the ambulance transfer time from scene to hospital in most of England is already short, this model is unlikely to be adopted in the NHS[17].

Feedback from the hospital team to paramedics about individual stroke assessments appears to improve future adherence to pre-hospital protocols including pre-notification[18] but no process exists to routinely facilitate this process. For unselected emergency admissions, a paramedic-initiated standardised communication approach appears to improve the accuracy and efficiency of handover[19] and there may be value in a format which is stroke specific. In acute medical settings there is increasing evidence that checklists are effective for improving patient safety and protocol adherence[20], and a paramedic protocol could include simple questions to prompt important hospital care processes (e.g. confirm that communication has been established with the stroke specialist).

This study will evaluate the clinical and cost effectiveness of a paramedic-initiated ambulance and hospital care pathway which seeks to facilitate clinical and radiological assessment of patients presenting with acute stroke symptoms in order to specifically reduce delays in the delivery of intravenous thrombolysis. Patient and professional views about the care pathway will also be described. This Paramedic Acute Stroke Treatment Assessment (PASTA) pathway consists of structured pre-hospital information collection, prompted pre-notification, structured handover of information in hospital, assistance with simple tasks during the first 15 minutes of hospital assessment, a checklist to confirm progress after 15 minutes and a paramedic request for feedback before departure. The pathway has been developed through systematic review of the literature regarding enhanced roles of paramedics, and developmental workshops with clinicians and support personnel in order to define professional roles and operational boundaries which are feasible whilst maximizing value for patient care.

6. Study aims and objectives

6.1 Aim

To determine the clinical-and cost-effectiveness of an enhanced Paramedic Acute Stroke Treatment Assessment (PASTA) pathway.

6.2 Objectives

- To determine whether the PASTA pathway improves patient care and outcomes. Primary outcome: proportion of patients receiving intravenous thrombolysis. Secondary outcomes: stroke severity 24 hours after thrombolysis (National Institute of Health Stroke Scale (NIHSS)[21]), complications after thrombolysis, inpatient mortality, discharge destination, discharge dependency (modified Rankin Score (mRS)[22]), assistance at discharge, 90 day dependency (mRS), 90 day destination, assistance at 90 days.

- To describe the impact of the PASTA pathway on time intervals from emergency call and hospital admission to first brain imaging, thrombolysis treatment (if given), ASU admission and formal assessment of swallowing safety.

- To describe the number and subsequent diagnoses of suspected stroke patients who travelled to hospital with a study paramedic but following assessment at hospital were not given a diagnosis of stroke (‘stroke mimics’).

- To determine the cost-effectiveness of the PASTA pathway relative to standard NHS stroke care.

- To report patient experiences during the PASTA pathway, including confidence in the care process and information received about diagnosis and treatment.

- To report professional views and perspectives (paramedics and hospital staff) of the acceptability and feasibility of the PASTA pathway in the clinical setting.

7. Study design

The study design is a cluster randomised controlled trial (RCT) with internal pilot phase and parallel process evaluation. Participants will receive either the PASTA pathway (intervention group) or standard stroke care (comparison group).

8. Study setting

The study will take place within ambulance services and a selected number of receiving hospital sites. Hospital sites within each region will be selected to represent a range of service designs and historical efficiencies in the provision of acute stroke care. All sites will already be receiving emergency stroke admissions and can provide 24 hour access to brain imaging and a stroke specialist opinion in order to make a thrombolysis decision. The research environment will reflect the local care pathway for patients with acute stroke symptoms and will include the scene of the incident, ambulance, ED, radiology department and ASU.

9. Randomisation

PASTA is a cluster RCT in which the unit of randomisation is an ambulance station or, in the case of large ambulance stations, an identifiable paramedic team within a station. Clusters comprise the paramedics based within teams or at stations. Randomisation will be performed prior to the start of the trial in each region. Within each ambulance service, ambulance stations or paramedic teams (according to local service configuration) which feed into a study hospital will be randomised to delivering the PASTA pathway or to continue with standard stroke care. All paramedics will be given the option of not assisting with the study.

Stations or teams within each service will be stratified according to size (categorised as small, medium or large according to the personnel and resources available), and distance from the nearest study hospital admitting stroke patients (distance categorised as near or far, reflecting the local geography of each ambulance service). The use of these stratifying variables will ensure that PASTA care paramedics (intervention) and standard care paramedics (control) are approximately equally matched by operational characteristics. The actual randomisations will be performed, documented and reported by two statisticians at Swansea University Clinical Trials Unit.

Stations and teams randomised to continuing standard care will be informed that there is an ongoing study of pre-hospital assessment for stroke patents, but not be given any further information about the intervention. For paramedics who are part of a station or team randomised to deliver the PASTA pathway, study specific training and documentation will be provided. Paramedics who opt out of the study or who work within a station or team that was not selected to be involved in the study will continue to deliver standard care.

10. Study treatments

10.1 PASTA pathway (intervention group)

Patients in the intervention group will undergo assessment by a paramedic who has received specific training and holds study documentation supporting the PASTA pathway. A summary of the PASTA pathway is shown in figure 1. It consists of the following stages:

i. Information: The paramedic will seek additional information at the scene which is routinely considered during thrombolysis treatment decisions, but is typically not obtained until after hospital admission. This will include:

- The presence of language (i.e. dysphasia) or visual (i.e. visuospatial) problems during a simple clinical examination, which may indicate a level of stroke severity more likely to be considered for thrombolysis treatment than FAST symptoms alone.
- Prescription of anticoagulant medication which would require additional urgent measurement of blood clotting indices before a thrombolysis decision could be made. This medication is also an additional indication for urgent brain imaging by itself, as a stroke due to haemorrhage would trigger urgent reversal its effects.
- A recent medical history of surgery or bleeding which might exclude thrombolysis treatment because of an increased risk of uncontrollable haemorrhage.
- Any previous medical history of transient ischaemic attack (TIA) or stroke, which could assist interpretation of brain imaging and specialist evaluation of the risk versus benefit of thrombolysis treatment.
- The current level of dependency according to whether the patient requires direct assistance with feeding or walking, in order to judge the value of administering thrombolysis treatment relative to the effects of the new stroke.

Within the pathway these information categories will be prompted by the acronym PASTA which represents: Plus dysphasia or visuospatial impairment; Anticoagulant medications; Surgery or other bleeding recently; TIA or stroke previously; Assistance needed daily.

ii. Pre-notification: During emergency transfer the paramedic will always be expected to attempt a pre-alert to the destination hospital in accordance with existing local arrangements. Although pre-notification is already a component of standard care, compliance is variable. Existing arrangements differ within regional ambulance services and individual hospitals e.g. direct telephone contact from paramedic, or via ambulance dispatch to ED or to ASU. The individual hospital response can vary according to the timing of the admission (e.g. the patient may be received by a specialist stroke team during weekday office hours and by ED clinicians at other times). The PASTA pathway does not change local processes but routinely prompts standard pre-notification.

iii. Handover: On arrival at the hospital's designated location for stroke admissions, the paramedic will provide a standardised handover of stroke-specific information (FAST, onset time, alertness and PASTA details) to the hospital team, indicating any items which were unavailable at the scene. The paramedic will ask the receiving hospital team whether it is possible to go straight to the CT scan if the radiographer is ready, and will remind them about ideal time targets for scan (<15 minutes) and thrombolysis treatment (<30 minutes). A member of the ambulance crew will assist the team with rapid registration of the patient's details on the hospital patient administration system. Prior to CT scan, a hospital clinician will always confirm that the provisional diagnosis is likely to be stroke within the previous 4 hours. In addition, the paramedic will inform the team about the known location of any relatives in order to facilitate information gathering, communication and treatment decisions. As shown in figure 1, the handover structure can be summarised by the abbreviation "FASTA PASTA CT".

iv. Scan: If the CT scan is immediately available, the paramedic will assist with patient transfer to the scan room accompanied by at least one member of the hospital team. It will be a local

decision whether the patient is first transferred onto a hospital trolley but any delays should be minimised.

v. Assist: If the CT scan is not immediately available, the hospital team will continue with urgent care of the patient according to the local service protocol and the patient will be transferred onto a hospital trolley. If ambulance service operational conditions are suitable and the hospital team require assistance, the paramedic can assist with the following tasks: insertion of an intravenous cannula if not already inserted, determining the patient's weight from assisting with transfer onto measurement scales or contributing towards a team estimation of weight, and repetition / clarification of clinical information (e.g. repeating the handover to a member of the stroke team). If the scan becomes available within 15 minutes of handover, the paramedic will assist with the patient transfer as above.

vi. Checklist: At 15 minutes after handover the paramedic will ask a member of the hospital team to confirm progress with key tasks: the status of the emergency brain scan request (if not yet performed) and stroke specialist review, confirmation of relevant medical history and medications, and ordering blood tests for clotting indices if relevant.

vii. Feedback: After completion of the checklist, the paramedic will request feedback from a hospital clinician about the provisional pre-hospital diagnosis of stroke, the estimation of onset time and any other aspect of the assessment process.

viii Completion: After seeking the checklist and feedback information the paramedic will complete and sign the study documentation. One copy will be retained at the hospital and a further copy will be returned to the ambulance service. The paramedic will depart as per usual operational procedure. This will be within 15-30 minutes of hospital arrival and therefore compliant with NHS Commissioning Board guidance for ambulance to hospital handover of emergency admissions[23].

The paramedic will record a reason if study documentation is signed before completion of the PASTA pathway. Anticipated reasons are:

- The paramedic is no longer contributing towards the clinical care of the patient.
- A change in the clinical state of the patient making the PASTA pathway no-longer appropriate (further details below).
- A stroke mimic condition is clearly identified during the initial hospital assessment and the hospital team determine that it would no longer be appropriate to continue with a care pathway for suspected stroke (further details below).
- A specific request by the regional ambulance control centre that the ambulance crew should become available for another call due to the pressure on resources.

Figure 1: PASTA pathway

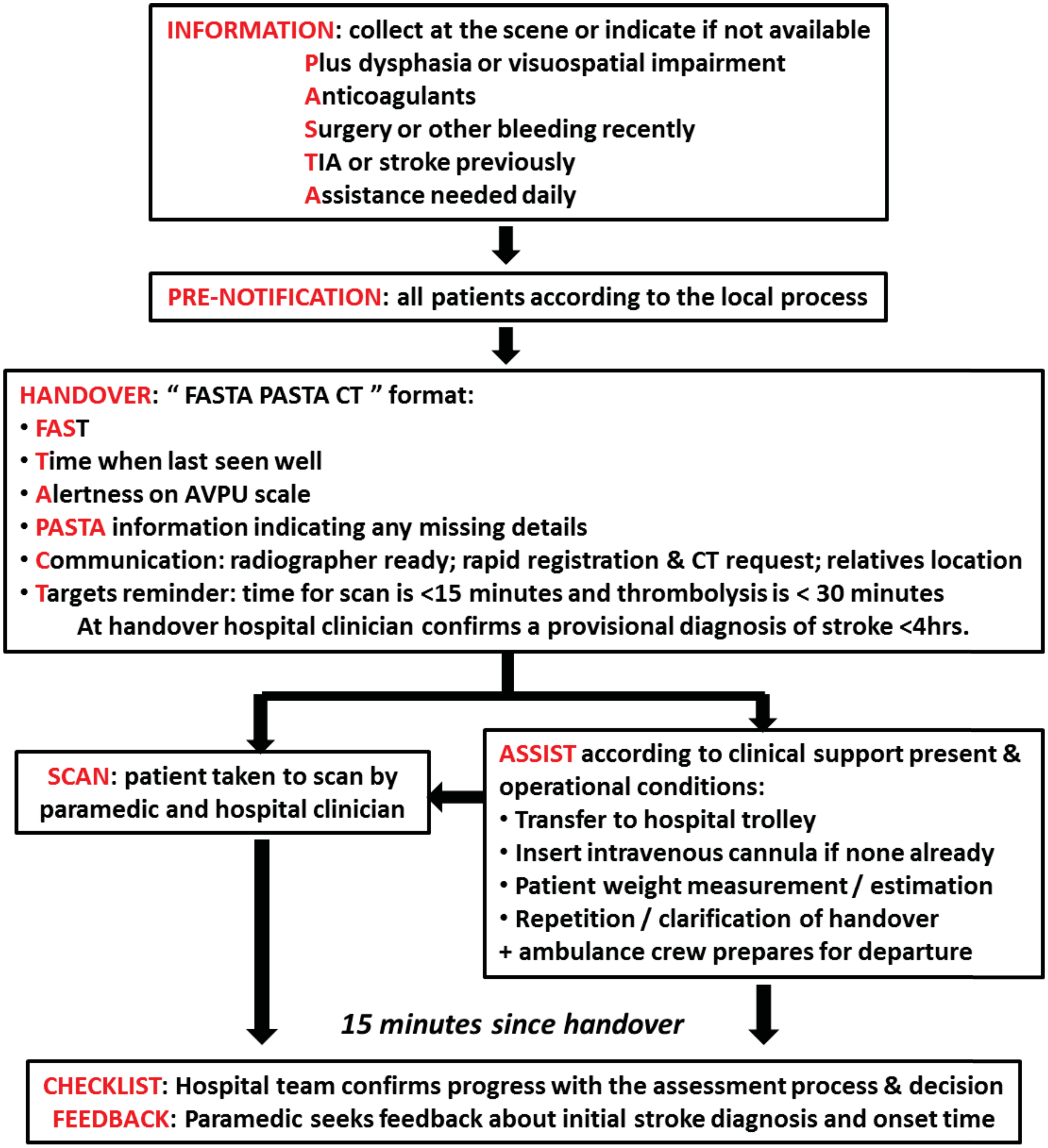

Permitted local variations to the PASTA pathway

Due to pre-existing variations in standard care clinical pathways and underlying healthcare service structures, there will be permitted variations in the different stages of the PASTA pathway as shown in the table below.

PASTA component	Core content expected to be delivered	Local variation permitted
Information	Must use PASTA format.	Any clinical information collection system (paper/electronic)
Pre-notification	Must be performed for all PASTA admissions.	Paramedic call to ED.<br>Paramedic call to ASU.<br>Dispatch call to ED.<br>Dispatch call to ASU.<br>Pre-notification may or may not include patient identifiable information.
Handover	Must follow FASTA PASTA CT format.	Occurs in most suitable area for rapid handover and registration.<br>Receiving hospital team are ED and/or stroke service clinicians.<br>May include other relevant information for individual patients.
Scan	Paramedic assists with transfer to scan if <15minutes since handover.	Any appropriate hospital clinician accompanies patient.<br>Any brain imaging modality which would assist treatment decisions.<br>Transfer by ambulance or hospital trolley as long as no delay incurred.
Assist	Patient transferred off ambulance trolley onto hospital trolley.<br>Paramedic offers assistance for initial tasks.<br>Ambulance crew prepares for departure.	Team may not require assistance for each or all of tasks.<br>Ambulance operational conditions may shorten paramedic stay.
Checklist	Paramedic enquires about progress from any hospital team member at 15 minutes since handover.	Checklist completion should reflect usual local service procedures (e.g. telemedicine specialist review)
Feedback	Paramedic seeks feedback about provisional diagnosis and onset time.	Any member of hospital team can provide feedback.<br>Paramedic can seek feedback on any aspect of the assessment.<br>The hospital team may not (yet) be able to provide feedback.

Clinical deterioration

During the pre-hospital phase of the PASTA pathway, if the patient’s condition deteriorates then the paramedic will re-evaluate whether it is still appropriate to continue with the pathway according to their professional judgement and standard clinical protocols. The reason for any deviation will be recorded including the anticipated clinical scenarios of falling conscious level, seizure, hypotension and hypoglycaemia.

Stroke mimic conditions

Early identification of stroke patients is challenging because other conditions (“stroke mimics”) can create similar symptoms through different mechanisms e.g. unwitnessed epileptic seizures and migraine. In clinical practice approximately 20% of patients who are FAST positive receive a stroke mimic diagnosis following admission to hospital, brain imaging and stroke specialist review. Thrombolysis treatment would be inappropriate. If a stroke mimic condition becomes apparent during the initial hospital review, the hospital team will discontinue the thrombolysis assessment process as per standard clinical care.

10.2 Standard stroke care (comparison group)

Patients in the comparison group will receive standard stroke care as per current local ambulance and hospital clinical protocols, which are reinforced by national clinical guidelines and audit. The study will not provide comparison group paramedics with additional training or documentation to support information collection, clinical communication or processes after hospital arrival.

The local thrombolysis assessment and treatment processes in hospital will not be deliberately altered. It is routine practice that all patients receiving thrombolysis are observed for at least 24 hours and undergo repeat CT brain imaging at 24-48 hours to exclude intracranial haemorrhage. Recognised complications of thrombolysis include allergic reactions (angioedema), extra-cranial haemorrhage and intracranial haemorrhage causing symptomatic neurological deterioration (defined as with an increase in NIHSS ≥4 points within 36 hrs of thrombolysis associated with parenchymal haemorrhage on repeat brain imaging)[24].

Whether or not they receive thrombolysis, all patients with acute stroke should be admitted to an ASU within 4 hours of hospital arrival in order to receive specialist assessment and care tailored to their individual needs. The overall length of stay in hospital reflects pre-morbid conditions and dependency, stroke severity and subsequent medical complications (e.g. pneumonia, thromboembolism, stroke recurrence), progress during rehabilitation, personal social circumstances and the local availability of services to provide further rehabilitation and care in the community (including generic and stroke-specific early supported discharge teams and community rehabilitation teams).

11. Study participants

Intervention group paramedics will be asked to deliver the PASTA pathway to the following patients:

- Aged 18 years and over.
- Face Arm Speech Test (FAST)[9] positive or any presentation of new focal neurological symptoms which indicate acute stroke in the paramedic's clinical judgement.
- Within 4hrs of last known to be without new stroke symptoms in the paramedic’s judgement.
- Admission to a study hospital

Patients will be identified and approached about enrolment in the study after arrival at hospital (see section 12 below). Patients approached about enrolment will meet the following criteria:

- Travelled to hospital with a study paramedic
- Aged 18 years and over
- Hospital specialist diagnosis of stroke
- Within 4 hours of stroke onset (onset time determined by the hospital stroke team) when assessed by the study paramedic

12. Participant identification, recruitment and consent

Patients will be identified, recruited and consented to take part in this study after arrival at hospital and when the thrombolysis treatment assessment has been completed. There will be no study enrolment process in the pre-hospital setting. The purpose of the study is to demonstrate that the PASTA pathway can expedite the clinical delivery of a treatment which is already known to be effective in reducing future disability, but must be administered rapidly (thrombolysis). A formal research consent process performed by study paramedics or the admitting hospital team would delay hospital admission, brain imaging and / or thrombolysis treatment. Due to the time-dependent effect of thrombolysis, even a short delay could reduce the impact of the PASTA pathway. As the pathway does not involve a new treatment or technology but is attempting to expedite an existing hospital care process using a structured clinical assessment performed by paramedics, the risk of harm to intervention patients is low. Patients will still only receive thrombolysis treatment following review by a stroke specialist. Although they will not provide study information to patients, as per usual clinical practice, paramedics will explain to patients about possible care processes which may occur in hospital.

In order to identify study eligible patients, hospital research support staff will systematically review the ambulance and hospital records of all admitted patients with a confirmed hospital specialist diagnosis of stroke. From the records and using the identification numbers of paramedics involved in the study, staff will identify those patients who travelled with a study paramedic, aged over 18 years and were assessed by the paramedic within 4 hours of the stroke onset time (onset time as determined by the hospital stroke team). Patients meeting these enrolment criteria will subsequently be approached about taking part in the study.

Ideally, approach of patients will take place during their inpatient stay such that a timely discussion about the study can be held. However, some patients are discharged very early after admissions and should identication of an eligible patient occur after discharge from hospital, a postal invitation to take part in the study will be used.

A site-specific participant eligibility log will be maintained.The participant eligibility assessment process is shown in figure 2.

Identification of confirmed stroke patients is facilitated by national clinical guidelines and audit which mandate that all stroke patients should be admitted to ASU within 4 hours of hospital arrival, irrespective of the timing or the local service configuration[5, 8]. It is still possible that a small number of stroke patients attended by a study paramedic will not be admitted to the local ASU because of:

- Transfer to a neuroscience centre for possible surgical or neuro-interventional treatment.
- Very quick recovery and discharge.
- Bed availability on the local ASU.
- Death before transfer from the ED to the ASU

In order to reduce the chance of these patients not being located in hospital, participating ambulance services will send a regular site-specific report of all suspected stroke patients admitted by study paramedics, to each participating site. For patients not listed on the site-specific participant eligibility log, hospital research support staff will check the hospital diagnosis assigned. Where the diagnosis is confirmed stroke, other study eligibility criteria will be reviewed and patients approached about the study as appropriate.

Patients whom the paramedic suspected to be suffering a stroke but who received an alternative diagnosis at hospital (‘stroke mimics’) will not be approached about study enrolment. However, the hospital diagnosis and any receipt of thrombolysis will be recorded to provide health economic context for the intervention. A site specific ‘stroke mimic log’ will

be maintained which will not contain patient identifiable material. Any study paperwork (e.g paramedic study documentation) generated by stroke mimic patients will be retained in the local investigator site file.

Figure 2: Identification of patients to approach for study participation

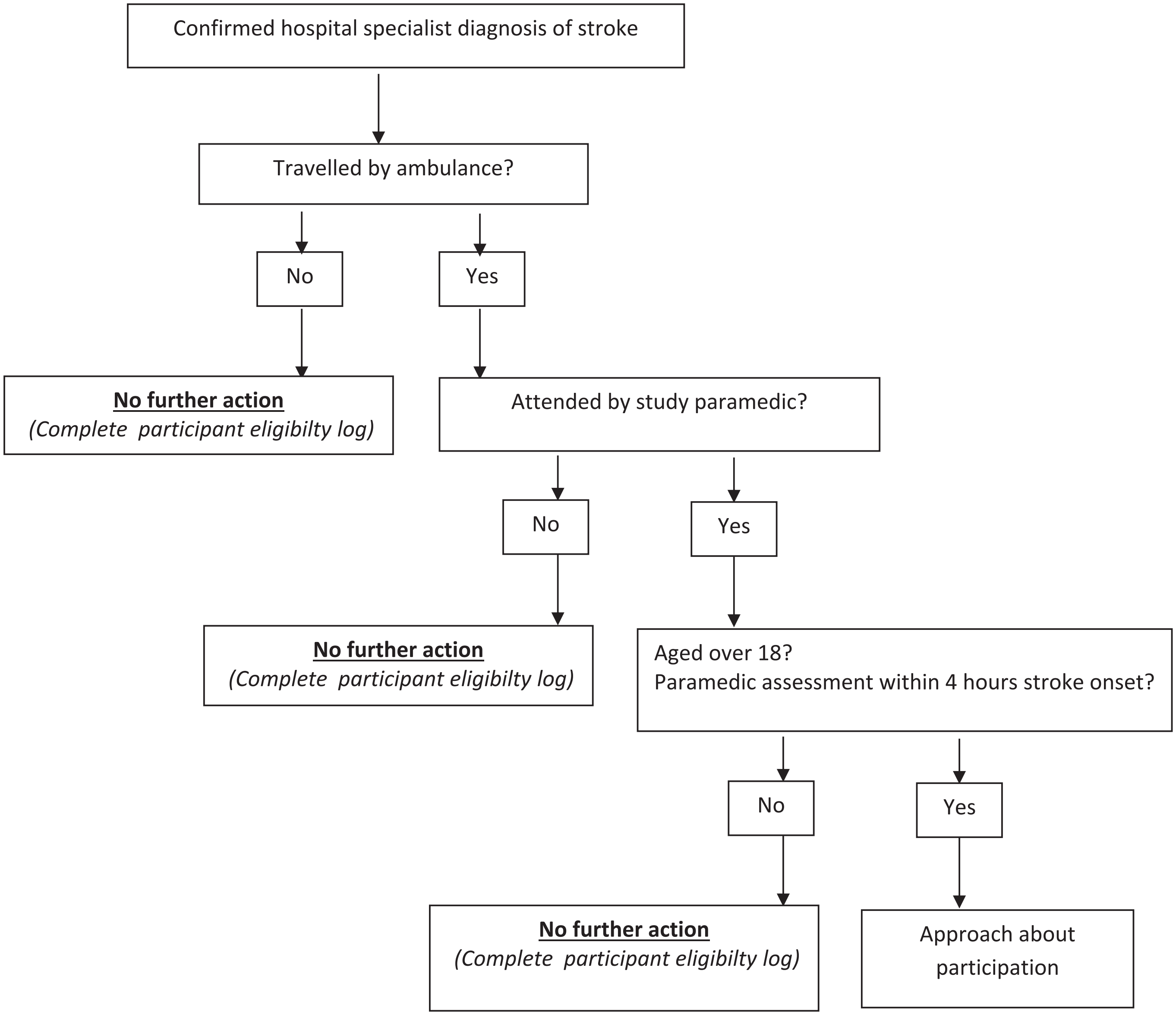

Consent

The consent process will seek permission for the use of routinely recorded healthcare data and for one study-specific assessment:

- Routinely recorded information from ambulance and hospital healthcare records will include: demographic details, stroke symptoms, physiological observations, relevant medical history and medications, CT scan result, thrombolysis assessment, thrombolysis outcome on day 2 (if relevant) and health status at discharge. In NHS stroke services these data items are systematically recorded for individual patients in healthcare records and reported publically at service level as a requirement of the mandatory prospective audit funded by the Department of Health i.e. the Sentinel Stroke National Audit Programme (SSNAP).

- The study-specific assessment will include collection of health outcome and resource utilisation information at 90 days after stroke. This will be conducted by face to face interview, telephone interview or postal questionnaire.

Consent for patients who can be approached about study participation during their inpatient stay

i. Consent for patients with mental capacity

For eliglble patients with capacity to consent to research, a trained member of the clinical team or NHS research support staff will approach the patient to discuss the study and provide a patient information sheet. After allowing sufficient time for potential participants to decide whether to take part in the study and an opportunity to ask questions, consent will be obtained in writing.

When a patient has mental capacity but is unable to sign the consent form (e.g. because of weakness of the dominant hand following stroke), consent will be confirmed orally in the presence of a witness (an individual not otherwise involved in the trial) and the witness will sign and date the consent form on behalf of the participant.

ii. Consent for patients with mild communication difficulties

For patients with mild communication difficulties due to the effects of stroke upon the use and understanding of language (aphasia), a set of ‘easy access’ study documentation will be used. After allowing sufficient time for the information (including an ‘easy access’ patient information sheet) to be considered and an opportunity to ask questions, consent will be obtained in writing using the ‘easy access’ consent form. NHS research support staff working within stroke services are experienced in communicating with patients with aphasia but specialist advice can be sought from the local stroke speech and language therapy service if required.

iii. Consent for patients who lack mental capacity

It is anticipated that approximately one third of study eligible patients will be unable to engage with an informed consent process due to the effects of stroke upon communication and cognition. As exclusion of this group would drastically reduce the clinical relevance of the study, if a patient has been identified as eligible but is unable to provide consent, a personal or professional consultee will be approached as detailed below..

For potential participants who lack capacity to consent to research, a trained member of the clinical team or NHS research support staff will attempt to identify an appropriate personal consultee (usually the next of kin) to approach, discuss the study and provide a consultee information sheet. If a personal consultee is identified, after allowing sufficient time for him/her to consider the patient’s wishes and feelings and an opportunity to ask questions, the consultee will be asked to complete a consultee declaration form if they believe the patient would have no objection to taking part in the study.

In the event of being unable to locate an appropriate personal consultee, an independent clinician (professional consultee) will be asked to confirm that the patient lacks capacity for consent, and that study participation would not introduce a risk of harm or be against the patient's wishes from what is known about their character and beliefs. The independent clinician will sign an independent clinician declaration form concerning study participation.

As it is likely that the communication or cognitive difficulties that impeded a patient's ability to provide consent will still be present at 90 days after stroke, where a personal consultee provided permission to enter the study, this person will be contacted to answer the 90 day health outcome questions on behalf of the patient. In cases where an independent clinician provided permission for study participation, only routinely available data will be collected at 90 days. The participant will not be contacted.

iv. ***Consent and early mortality***

The early mortality rate following acute stroke is approximately 10%. These patients are usually identified on admission and treated palliatively. However, unexpected deaths also occur. Exclusion of patients that die soon after admission would reduce the study's relevance for the typical clinical stroke population.

When a patient has died or if a formal palliative end of life care process has been started at the point when the patient is identified as eligible for the study, individual patient consent will not be possible and it is likely to be distressing for a personal consultee to be approached regarding the research use of routinely collected healthcare data. The 90 day health outcome will not be relevant. Under these circumstances the local PI will sign an Early Mortality / Palliative Care Declaration Form to confirm that the patient has died or is in a formal palliative phase, and take responsibility for the use of routinely collected healthcare data for this research project.

Acute stroke has many effects upon neurological function and consciousness which can fluctuate for several days or even weeks. There may be unusual instances where a patient who has been entered into the study using this Early Mortality/Palliative Care Declaration form subsequently shows enough signs of improvement that supportive care is re-instated, and may still be alive at day 90. However, these are likely to be challenging clinical situations where seeking an alternative method of consent will be difficult because of the severe degree of remaining neurological impairment and time elapsed since admission. In these unusual scenarios, an alternative method of consent will not be pursued and the day 90 study specifc assessment will not be conducted. Routinely available data collected as part of standard clinical care will be retained as per the original PI declaration and used in the analysis.

v. Loss of capacity to consent to research during participation in the study

When participants have provided their own consent to take part in this research project, it is possible that they may temporarily (e.g. because of intercurrent illness) or permanently (e.g. because of further stroke) lose the capacity to assist with the 90 day outcome assessment. On entering the study, participants will be asked to nominate a personal consultee (relative or close friend) who may be contacted to answer the 90 day health outcome questions on their behalf, should they be unable to undertake this assessment personally.

Consent for patients who are only identified as study eligible after discharge from hospital

Patients who have only been identified as study eligible after hospital discharge will receive an invitation letter, patient information sheet, consent form and pre-paid return envelope by post. Patients willing to take part in the study will be asked to return a completed consent form. Invited patients who have not returned a consent form within four weeks will receive one phone call from the local hospital research team. Therefter, patients will only be further contacted about the study if a consent form is returned,

Figure 3 summarises the decision process for obtaining study consent,

Consent forms

Original consent forms will be retained in the investigator site file at each study site. A copy of the form will be filed in the medical records, with a further copy given to the participant. For participants returning the consent form by post, a copy of this will be returned to the participant along with a letter acknowledging receipt and study participation.

The information sheets and consent forms will be available in English. However, interpreters and translation of written material will be possible through local NHS arrangements should potentially eligible patients require this.

Figure 3. Decision process for study consent

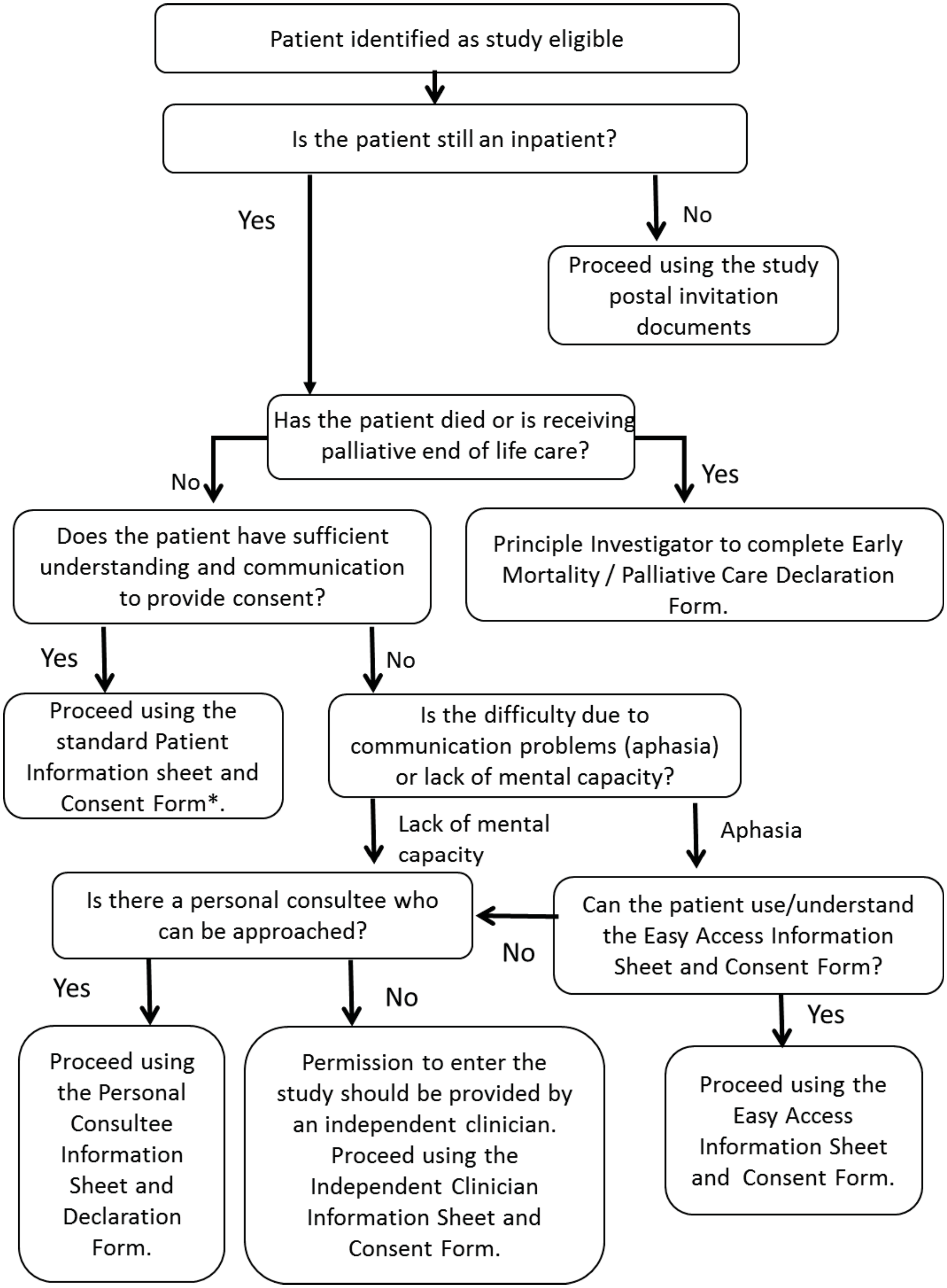

**When a patient has mental capacity but is unable to sign the consent form (e.g. because of weakness of the dominant hand following stroke), consent may be confirmed orally in the presence of a witness (an individual not otherwise involved in the trial) using the box at the bottom of the standard consent form.*

13. Study data collection

Data for this study will be collected day 1 pre-admission, day 1 post-admission, day 2 (if thrombolysis administered) and 90 days (+/- 7 days) post stroke.

Day 1 pre-admission data:

Suspected stroke diagnosis (FAST; paramedic judgement of stroke or query stroke)
Symptom onset or last known to be well time (paramedic judgement)
Pre-notification of receiving hospital
Times of 999 call, ambulance on scene, ambulance left scene and hospital arrival
Involvement of rapid response paramedic: yes/no (invention group only)
Time of transfer of care (also called handover) from the paramedic to the hospital team
Time of paramedic signature of the completed ambulance clinical record
Time paramedic is clear to respond to another incident

The above data will predominantly be transcribed from the routine ambulance clinical record onto a study specific Case Record Form (CRF) by NHS research support staff or other hospital clinical staff trained to deliver this research project. However, some data items are not contained within the routine ambulance clinical record but held within computerised ambulance service dispatch systems. These data will be obtained directly from the ambulance services. For participants travelling to hospital with an intervention paramedic trained to deliver the PASTA pathway, some of the above data will also be recorded onto a study specific paramedic CRF which will additionally record delivery of the PASTA pathway.

Day 1 post-admission data:

Demographic information (age; gender; pre-stroke mRS)
Date/time of hospital admission
First blood pressure reading on admission
First blood glucose reading on admission (capillary or serum glucose)
Stroke severity on admission (National Institute of Health Stroke Score)
Stroke onset or last known to be well time (stroke team judgement)
Current use of anticoagulant medication
Previous medical history (stroke; TIA; heart failure; atrial fibrillation; diabetes; hypertension)
Hospital admission locations (ED; stroke unit; critical care; medical admissions ward, other ward)
First brain imaging time, modality and result (CT / MRI; infarction/primary intracerebral haemorrhage)
Thrombolysis treatment decision (yes / no; reason why thrombolysis not administered)
Date/time of thrombolysis (if received)
Use of other treatments (reversal of anticoagulation if haemorrhagic stroke; acute blood pressure lowering if haemorrhagic stroke or before intravenous thrombolysis; referral or transfer to neurosurgery; referral or transfer for intra-arterial treatment; use of intra-arterial treatments (yes / no; time of puncture)).
Entry into another clinical trial on day 1 after admission (yes / no; which one)
Date and time of admission to first stroke unit
Date and time of assessment of swallow safety at first hospital

Day 2 thrombolysis outcome (if administered):

Stroke severity (NIHSS) at 24-48 hours after thrombolysis treatment (and/or intra-arterial treatment if received): standard post thrombolysis stroke severity measurement.
Complications (symptomatic intracranial haemorrhage / extracranial bleed / angioedema / other complication) within 48 hours of thrombolysis treatment (and/or intra-arterial treatment if received): standard post thrombolysis complications measurement.

The post admission data are all routinely collected clinical data. They will be transcribed from routine medical records onto a study specific hospital CRF. This will be performed by NHS research support staff or other clinical staff trained to deliver this research project.

Day 90 (+/- 7 days) data:

At day 90, both data related to hospital discharge from this continuous inpatient episode and health at 90 days after stroke will be collected.

Data related to discharge due to completion of all inpatient care:

Died during this inpatient episode for stroke (yes/no)
Cause of death
Date of death (if relevant)
Discharge date or record that the patient is still an inpatient at day 90 (yes/no)
If still an inpatient at day 90, has there been stroke recurrence
Discharge destination at completion of this inpatient episode (own home, different private address (eg relative) care home, other)
If discharged to a care home, was the patient previously resident / not previously resident
Rehabilitation arranged at discharge (none, early supported discharge team, community rehabilitation team, both)
Assistance with activities of daily living required at discharge (yes,/no; if yes: what support received: informal carers/paid carers/both)
If paid carers then how many visits per week provided at discharge
Dependency at discharge (mRS)

Data related to day 90 health:

Died after stroke event discharge but prior to 90 days (yes/no)
Date of death (if relevant)
Cause of death (if available)
Readmission(s) to hospital since discharge; reason for admission(s), length of stay(s).
Readmission was due to stroke recurrence (yes, no)
Current (90 day) residence (hospital, home, different private address, care home, other).
Current (90 day) dependency (simplified questionnaire mRS[25])
Community rehabilitation since discharge (therapy received (yes/no); received at home/hospital/both; number of weeks received; less than, equal to or great than one appointment/week).
Current (90 day) assistance with activities of daily living (yes / no; if yes: what support received: informal carers/paid carers (how many visits per week) /both)

For all participants, confirmation that stroke remains the diagnosis assigned for the event where consent was obtained. If not, the new diagnosis assigned.

Day 90 data consists of both routinely collected clinical data and study specific data. Routinely collected clinical data will be transcribed from routine medical records onto a study specific CRF. Study specific data will be collected by face to face interview, telephone interview or by postal questionnaire. Telephone interview with the participant or consultee will be the main method, undertaken by NHS research support staff.

In instances where a participant/personal consultee cannot be contacted by telephone after one week of attempting to locate him/her, a postal questionnaire will be used. A postal questionnaire will also be used where telephone interview is not be possible (e.g. participant does not have a telephone). A single postal questionnaire with enclosed reply-paid envelope will be mailed. It will also be possible to collect 90 day study specific data by face to face interview conducted by NHS research support staff. This option will be used at the discretion of staff but predominantly when participants are currently hospital inpatients.

Where it has not been possible to obtain the 90 day study specific data by 6 months after stroke, NHS research support staff will use the routine hospital data administration systems to re-confirm whether the participant was alive or dead at 90 days. This is necessary because deaths in the community can take some time to appear in the routine hospital records. The mRS (primary outcome measure) is scored 6 for death[26].

All study data will be entered locally onto a secure online database maintained by Newcastle Clinical Trials Unit. Pseudo-anonymised participant identification codes will be used. Study paper CRF's will be kept securely in the local investigator site file.

14. Blinding

In this trial, no specific blinding measures will be in place. Paramedics will know the study group allocation due to the nature of the intervention. Participants will be informed about group allocation if they ask for this information. NHS research support staff who will conduct the 90 day research assessment may be unaware of treatment group allocation but this will vary according to their involvement with participants on admission to hospital.

15. Staff training and awareness

For paramedics based within stations / teams randomised to deliver the PASTA pathway, study specific training will be provided via a securely hosted online video. This will include explanation of the study objectives, demonstration of the PASTA pathway processes, completion of trial documentation and responses to adverse events. After watching the video, paramedics will complete an online MCQ knowledge assessment. Paramedics who do not provide correct responses to all questions will be invited to watch the online video again and repeat the MCQ. Paramedics who correctly answer all questions will be identified as trained and issued with study documentation. If paramedics based with stations / teams allocated to delivering the intervention do not wish to assist with the study, they will be able to opt out of training.

Paramedics based with stations / teams randomised to continuing standard stroke care will be informed that there is an ongoing study of pre-hospital assessment for stroke patents, but not be given any further information about the study and asked not to change their practice. Paramedics allocated to continuing standard stroke care will also be able to opt out of the study if they wish.

To determine whether the characterisitcs of paramedics participating in each study group differ, the following will be obtained about individual randomised paramedics during the course of the study: age, gender, highest qualification, years qualified as a paramedic, years in service at employing ambulance service, NHS job band, job title. These characteristics will be obtained from ambulance service records if possible and if not, direct from paramedics by means of an electronic survey.

NHS research support staff based in hospitals taking part in the study will receive study specific training. This will cover identification, recruitment and consent of participants, data collection and the 90 day research assessment, and other study processes such as use of the online database.

Clinical staff at hospitals taking part in the study will be made aware of the trial at service meetings and by email, but will not receive additional protocol-related research training or documentation. Hospital services will not be expected to change their standard local care process for patients undergoing thrombolysis treatment assessment as part of the study. They will continue to provide clinical care for all study patients consistent with the information and prompts provided by all paramedics within the context of the local service setting. If services change their configuration and standard care process during the study as a quality

improvement initiative, this would apply to all stroke admissions and would not prevent the delivery of the paramedic-led PASTA intervention.

16. Study withdrawal

No specific withdrawal criteria have been pre-set. Participants may withdraw from the study at any time for any reason. Data collected prior to withdrawal will be used in the study analysis unless the patient or their representative requests that this should not be the case. Should a decision to withdraw from the study be made, a reason for withdrawal will be sought but participants can chose to withdraw without providing an explanation.

Occasionally, further clinical tests and information obtained result in a diagnosis of stroke being revised to an alternative diagnosis at a later date. Such patients will not be withdrawn from the study as initial care processes received were for a diagnosis of stroke.

17. Safety evaluation

The standard definitions for adverse events will be used in this study:

Adverse event (AE): Any untoward medical occurrence in a subject to whom a study intervention or procedure has been administered, including occurrences which are not necessarily caused by or related to that intervention. An AE, therefore, does not necessarily have a causal relationship with the treatment. In this context, "treatment" includes all interventions (including comparative agents) administered during the course of the study. Medical conditions/diseases present before starting study treatment are only considered adverse events if they worsen after starting study treatment.

Related AE: An AE that results from administration of any of the research study procedures. All AEs judged by either the reporting investigator or the sponsor as having reasonable causal relationship to a study procedure qualify as 'related adverse events'. The expression "reasonable causal relationship" means to convey in general that there is evidence or argument to suggest a causal relationship.

Causality: The assignment of the causality should be made by the investigator responsible for the care of the participant. All adverse events judged as having a reasonable suspected causal relationship to a study procedure are considered to be related adverse events. If any doubt about the causality exists, the local investigator (PI) should inform the Chief Investigator. In the case of discrepant views on causality between the investigator and others, all parties will discuss the case. In the event that no agreement is made, the main REC and other bodies will be informed of both points of view.

Serious Adverse Event (SAE): an untoward occurrence that:-

- Results in death
- Is life-threatening (refers to an event in which the subject was at risk of death at the time of the event; it does not refer to an event which hypothetically might have caused death if it were more severe)
- Requires inpatient hospitalisation, or prolongation of existing hospitalisation
- Results in persistent or significant disability or incapacity
- Consists of a congenital anomaly or birth defect
- Is otherwise considered medically significant by the investigator

Medical judgement should be exercised in deciding whether an AE is serious in other situations. Important medical events that are not immediately life-threatening or do not result in death or hospitalisation but may jeopardise the patient or may require intervention to

prevent one of the other outcomes listed in the definition above, should also be considered serious.

Unexpected Adverse Event: An adverse event that is not an expected occurrence in the circumstances of this trial.

Expected adverse events

The PASTA pathway is embedded into standard clinical practice for a clearly defined time interval, during which patients will be closely supervised by qualified ambulance and hospital professionals. Deterioration in the health status of stroke patients commonly occurs in clinical practice: average inpatient mortality is 17% in NHS services[5]; 2% patients treated with thrombolysis deteriorate due to the recognised side effect of cerebral haemorrhage[6]; the stroke recurrence rate is up to 6% within the first 3 months[27]. The following are therefore expected adverse events:

Death directly due to the initial stroke.
Thrombolysis related cerebral haemorrhage.
Stroke recurrence leading to new hospitalisation.

Recording and reporting of adverse events

This study will only report adverse events which are considered to be serious.

In addition, apart from death, adverse events which are considered to be serious will only be reported up until seven days after study entry (ie for one week after the date of the paramedic assessment).

After seven days, there is no possible mechanism by which the study intervention can cause new adverse events. This approach mirrors other clinical trials of emergency stroke treatment where the longer term risk of unforeseen adverse events from the study intervention is very low. Any adverse events that do occur will be in the immediate peri-admission period as the intervention has been designed to facilitate the emergency administration of intravenous thrombolysis to appropriate stroke patients. Thrombolysis treatment is widely used in clinical practice and its adverse events are already well described. It can only be given once within 4.5 hours of stroke onset and has lost all pharmacological action within 24 hours. The study intervention does not change the clinical criteria for thrombolysis administration. Therefore it is not possible for any adverse events after the emergency phase to be attributed to the study intervention. To provide additional reassurance, the reporting period has been set at 7 days rather than just 24 hours.

Deaths will be recorded for the duration of a participants involvement in the study (until 90 days after stroke).

Serious Adverse Events will also **exclude**:

- The expected adverse events listed above (these will be routinely recorded and monitored as part of study data collection)

- Pre-planned hospitalisations and scheduled treatment for pre-existing conditions.

A study SAE form will be completed for all SAEs. To ensure complete capture of SAE's, the 90 day study CRF will include a question to ask staff to check for medical events that fulfil the criteria to be a SAE. For any events which fulfil the above criteria to be a SAE and are unreported, the study SAE form will be completed.

Serious adverse events, regardless of causality or expectedness, will be reported to the Chief Investigator. SAEs will also be reported to the trial sponsor (Newcastle Upon Tyne

Hospitals NHS Foundation Trust) in line with sponsor policies. The initial report can be made by telephone or email to the study co-ordinating centre. All provisional reports must be followed by a fully completed form. If incomplete information is available at the time of this initial report, further information must be provided on a follow up form as soon as it is available. The main REC will be notified of related and unexpected SAEs within 15 days of the Chief Investigator becoming aware of the event.

18. Statistical analysis

Analysis is by ‘treatment allocated’; that is, primary and secondary efficacy and safety outcomes analyses will all use the study group allocation of the attending paramedic; only paramedics completing the study specific training will be considered as members of the intervention group. Thus, data will be analysed pragmatically according to paramedic group allocation, irrespective of the care process actually experienced by individual participants.

The primary end point measure is the proportion of participants who received intravenous thrombolysis. The primary analysis will compare the proportion of participants who received thrombolysis in the two arms of the study using logistic regression. Models will include various covariates and factors, including (but not limited to) participant age and gender and admission time and date (used to define a covariate to reflect background trends).

Secondary outcomes, including stroke severity 24 hours after thrombolysis (measured by an NIHSS score), inpatient mortality and dependency (modified Rankin Scale), will be analysed by appropriate ordinal and binary regression models, with appropriate adjustment for potential covariates and factors.

To address the second objective (that is, the impact of the intervention on various time intervals related to the emergency call), we will use appropriate statistical models. For time intervals between events which always happen (for instance, emergency call to hospital admission), we will employ linear models (after appropriate transformation), again adjusting for statistically significant covariates and factors; if the distributions of residuals are markedly non-normal, further transformation or bootstrapping will be considered. Residual diagnostics will also be used to identify outliers; identified outliers will be excluded and the analysis recalculated. For events which may or may not happen (for instance, emergency call to thrombolysis), we will analyse outcomes via binary regression models and survival analysis, comparing intervention and control arms using the framework of accelerated life testing; both approaches will again accommodate statistically significant covariates and factors.

Other clinical objectives will be addressed by appropriate descriptive statistical measures.

Sensitivity and subgroup Analyses
We will undertake various sensitivity and subgroup analyses to supplement evidence from the primary analysis to help to characterize fully the treatment effect. These will include (i) analysis by ‘treatment received’; (ii) analysis of the study subgroup with a diagnosis of stroke unchanged at 90 days; (iii) others. Subgroup analyses will generally, but not exclusively, be undertaken for specific categories in factors with a significant relationship with the primary outcome measure. These analyses are either confirmatory or exploratory and, as the study is not statistically powered for multiple analyses, their results will be interpreted in this context.

Missing Data
In general, we shall endeavour to adopt a consistent approach to missing data relating to both clinical and cost effectiveness, except where individual outcome measures require variation in that approach. For each variable, we shall summarise the frequency of missing data, which affects effective sample size and hence statistical power. If there is no reason to suspect that data are not Missing Completely At Random (MCAR), we shall consider the use

appropriate imputation methods to ameliorate the problem of missing data; otherwise, the Trial Statistician and Chief Investigator will further discuss patterns in missing data.

Reporting
Outcome descriptions, summaries and comparisons will be expressed in accordance with appropriate CONSORT guidelines[28], including estimates with 95% confidence intervals to summarise two-tailed tests at the 5% significance level.

Sample size

Sample size considerations are based on postulated and standardised statistical effects of the intervention on binary outcomes. A change from 0.1 to 0.13 (for instance, in the proportion of cases with a good mRS score at day 90) is equivalent to an effect of 0.1, while a change from 0.43 to 0.53 (for instance, in the proportion of patients receiving thrombolysis) is equivalent to an effect of 0.2.

In the initial protocol for this trial, sample size considerations assumed equal patient numbers per arm, allowed for 3% attrition for a primary outcome collected at day 90, used conventional values of 80% power and 5% signficiance, and a relatively small change between arms from 0.1 to 0.13 in the proportion of cases with a good mRS score at day 90, equivalent to a standardised statistical effect of 0.1. These considerations led to an initial recruitment target of 3640 (1820 cases per arm).

However, in early 2017, informal review of internal phase data showed that some key assumptions were unlikely to be met in practice. Specifically, overall recruitment was lower than anticipated, while intervention paramedic training completion rates meant a persistent imbalance in patient numbers in the two arms, with typically 8-10 control cases for every 5 intervention cases.

Following discussions with stakeholders (including both trial oversight committees and funders), it was agreed to amend the primary outcome to the proportion of patients receiving intravenous thrombolysis; postulating a change from 0.43 to 0.53 in the proportion of patients receiving thrombolysis, equivalent to a standardised statistical effect of 0.2. Sample size considerations were therefore re-assessed, factoring in attrition of 1%, power of 90% (and 5% significance), and an average cluster (patients per paramedic) of 5 with an intra-cluster correlation coefficient of 0.02. In addition, an imbalance of 2 control cases per intervention cases was incorporated. This gave a revised recruitment target of 1297 study participants (865 control cases versus 432 intervention cases).

This revised target sample size should, if underlying assumptions are satisfied, lead to 1284 analysable outcomes (856 control; 428 intervention), However, should the attrition rate or imbalance ratio change during the course of the study, it may be necessary to adjust the final sample size accordingly to achieve the required number of control and intervention analysable outcomes needed to maintain statistical power.

19. Internal pilot study

The study will commence as an internal pilot trial involving the North East Ambulance NHS Foundation Trust and two North East hospital sites. The success of the pilot study will be judged against the following criteria after 4 months of recruitment activity:

1. > 80% of confirmed stroke admissions are assessed for study eligibility. This will be determined by comparison of the participant eligibility log data and data extracted from routine hospital systems which record stroke admissions.

2. >80% of patients identified as eligible for the study are approached about study participation.

3. Each hospital site has recruited a minimum of 4 participants/month into the study. This number is lower than an estimated predicted monthly recruitment rate for each hospital, however, the uniqueness of this study makes it difficult to precisely predict a recruitment rate until the study commences.

4. Of the participants recruited to the study who travelled with an intervention paramedic, >50% had the PASTA pathway information handover attempted.

If any of these criteria are not achieved, a review of the appropriate study procedures will be undertaken to determine why it was not possible for a criterion to be achieved. If it is believed that a change to processes will enable the study to be delivered, changes will be made and the study will continue. Decisions will be discussed and agreed with the Trial Steering Committee.

If any of these criteria are not achieved, a review of the appropriate study procedures will be undertaken to determine why it was not possible for a criterion to be achieved. If it is believed that a change to processes will enable the study to be delivered, changes will be made and the study will continue. Decisions will be discussed and agreed with the Trial Steering Committee.

20. Economic analysis

The primary economic analysis will be a within trial economic evaluation to estimate cost-effectiveness of the enhanced role versus usual care in increasing the proportion of patients receiving intravenous thrombolysis, and will report a cost per additional patient thrombolysed. In addition a cost utility analysis will be conducted to estimate cost per quality adjusted life years (QALYs).

To derive health outcomes at 90 days, a model based simulation will be constructed using a model previously developed which describes outcomes at 90 days based on whether or not thrombolysis was given, time elapsed since stroke onset if it was given and other physiological and demographic factors[29]. A Monte Carlo approach will be take in which different subsets of trial participants from each arm are repeatedly sampled and their health status at 90 days is estimated. The effectiveness and cost-effectivessness of the enhanced role versus usual care will be the average of all the samples, together with an estimate of uncertainty.

Pre-stroke and 90 days mRS scores will be used to estimate QALYs using mapping algorithm which has been developed by Rivero-Arias et al. to estimate the EQ5D scores from mRS values[30]. Mortality and utility values will be converted into QALYs using the area under the curve method, controlling for baseline health utility derived from mRS[31]. The uncertainty analysis results will be presented as a cost effectiveness acceptability curve (CEAC)[32]. The CEAC will show the probability that an enhanced role is cost-effective

compared with usual care, given the observed data, for a range of maximum monetary values that decision makers may be prepared to pay for percentage change in proportion of patients that are thrombolysed /extra QALY.

Estimating the resources used and costs of providing an enhanced paramedic role:

The costs of paramedic time will be based on the equivalent hourly wage for the appropriate grades. The time taken to complete the training will be recorded by the computer based training software. Training material productions costs will be excluded from the evaluation. The times from the “on scene” to “handover” and “clear to respond to another incident” will be recorded and used to estimate additional ambulance service resource utilisation that may result from the enhanced paramedic role. The costs of these resources will be obtained from Ambulance Trusts and published sources including the PSSRU.

Estimation of consequences for resource use of an enhanced paramedic roles resulting from improved stroke outcomes:

Data on the use of NHS inpatient resources used will be collected for patients in both arms of the trial. This data will be comprised of brain imaging modality, provision of thrombolysis and length of stay in hospital. In addition, data about community rehabilitation therapy, social service involvement and non elective episodes of secondary care post discharge will also be collected. This information will be collected as part of the study day 1/2 and day 90 data collection points.

Unit costs will be derived from routines sources for NHS and social care. The costs of providing the PASTA pathway for stroke mimic patients will be included within the economic evaluations but also presented separately. This will represent an economic worst case scenario because costs will be added but it will be assumed that there is no benefit (or harm) to these individuals. If the intervention still appears cost-effective in this scenario then the trial conclusions would be strengthened.

Missing resource use data:

Where there is data missing about post acute resource usage, essential items will be imputed from data present in the study for similar patients as defined by age, stroke severity and 3 month outcome[33]. Sensitivity analysis will quantify the degree of bias introduced by imputation.

Sensitivity Analyses:

Sensitivity analyses will be conducted on key parameters and assumptions contributing the estimates of cost-effectiveness. They will be designed to estimate the robustness of the findings and to provide additional information to decision makers. For example, how estimated cost-effectiveness is affected by assumptions about the extent of the resource and financial burden on ambulance services that result from paramedics performing an enhanced role.

Long-term economic evaluation:

In common with other economic evaluations, a further modelling analysis will be undertaken to extrapolate the results of the trial beyond the 90 days, which will help to inform likely longer-term cost and effects. The model will be a microsimulation state transition model made up of three states (independent, dependent and death) to which costs and QALYs will be attached[34]. To estimate the longer term effects of increased independence that may result from an enhanced paramedic role in the timely treatment of stroke, the economic model will describe changes in dependency status over time. Outcomes after subsequent strokes (dependency measured by the modified Rankin Score at day 90) will be estimated

using a published decision analytic model (DAM) which predicts the outcome of treating acute stroke with and without thrombolysis[29].

Quantifying uncertainty in estimates of cost effectiveness:

Bootstrapping will provide estimates of the imprecision surrounding estimates of costs and incremental cost per unit of outcome. Results will be presented as cost effectiveness acceptability curves (CEACS)[32]. Probabilistic sensitivity analyses will be carried out on key cost parameters. If after initial analysis it is found that the cost-effectiveness of the intervention is sensitive to one or more inputs, an Expected Value of Perfect Parameter Information (EVPPI) will be undertaken focusing on those inputs[35].

21. Qualitative evaluation

A qualitative process evaluation will be undertaken during the trial to report upon patient experience and professional views (paramedics and hospital staff) regarding the acceptability and feasibility of the PASTA pathway in the clinical setting.

Four groups of participants will be purposively sampled and invited to participate in semi-structured interviews:

1. Medically stable patients <u>within the North East</u> region who were attended by an intervention paramedic and provided their own consent to participate in the pilot or main trial.

Participants with a range of stroke severities and treatment experiences will be recruited. Individual face to face interviews will be conducted. Whenever possible, interviews will be arranged within 7 days of admission to facilitate recall of experiences and details. Interviews will last up to 30 minutes. Participants enrolled in both the internal pilot trial (up to 12) and main trial (up to 40) will be included. The number of North East hospital sites where patients will be identified for invitation will be determined during the trial according to parameters such as recruitment rate and different stroke service design.

At agreed time intervals during the trial recruitment period the researcher conducting the interviews will make contact with a member of the research support staff at a chosen North East hospital site. Without receiving identifiable information, the researcher will enquire about the demographics (e.g. age, sex, stroke severity, thrombolysis received) of the participants who are allocated to the intervention group, received the PASTA pathway and who gave their own consent to take part in the trial in the previous seven days. Using the demographics, the researcher will select the participants to be invited for interview. A member of the research support staff will subsequently approach the nominated participants to discuss the interview process and provide an information sheet. After allowing sufficient time for participants to decide whether to take part in the interview and an opportunity to ask questions, the member of the research support staff will obtain consent to provide their contact details to the researcher conducting the interview.

The researcher conducting the interview will arrange a mutually convenient time to visit the patient (either in hospital or in their own home if recently discharged) to conduct the interview. Consent to be interviewed will be obtained immediately prior to the start of the interview (in writing).

2. Medically stable patients <u>outside of the North East</u> region who were attended by an intervention paramedic and provided their own consent to participate in the pilot or main trial.

Participants with a range of stroke severities and treatment experiences will be recruited. Telephone interviews will be conducted. As it would be logistically challenging to arrange these interviews within 7 days of admission, recruited participants may be approached at any time during their involvement in the trial (up to 90 days). Interviews will last up to 30 minutes. Only participants enrolled in the main trial will be included as sites outside the North East are not participating in the internal pilot trial. The number of participants to be included and the number of hospital sites where participants will be identified for invitation will be determined according to the progress of the trial.

The researcher conducting the interviews will identify participants to be invited for interview using the pseudo-anonymised data entered onto the study database (e.g. hospital site, intervention group, consent mode, age, sex, stroke severity, thrombolysis received). The researcher will subsequently make contact with a member of the research support staff at the hospital sites and ask them to mail invitation letters to the identified participants. Invitation letters will contain an enclosed patient information sheet, a self completion 'interview contact details' form and a pre-paid envelope for participants to return directly to the researcher if they are interested in taking part in the interview.

Upon receiving a completed 'interview contact details' form, the researcher will telephone the responding participants and explain the purpose of the interview. If the participant is willing to be interviewed, the researcher will arrange to mail/email a consent form and arrange a mutually convenient time for the telephone interview to take place. Prior to the conducting interview, the researcher must be in receipt of the signed consent form.

3. Paramedics

Following the start of this research project, the scope of paramedic interviews has been amended. The first study protocol detailed interviews with only intervention group paramedics who had completed the study training. However as a significant proportion of paramedics randomised to the intervention group did not undertake the training, the process evaluation has become an important opportunity to understand barriers and facilitators to the deployment of the intervention if it was found to be of value for patient care, and about pre-hospital research in general.Therefore interviews have been widened to include intervention group paramedics who have not completed study training after a reasonable amount of time despite repeated invitation and opportunity to do so.

For intervention group paramedics who have completed training, purposive sampling will aim to ensure representation of a range of times since PASTA pathway activation (less or more than 30 days; no activations since training); paramedic seniorities (years of practice; employment title); base station setting (urban; rural); and experience of different patient admission routes (via ED; direct to stroke unit). These data are collected as part of the study or from individual paramedics during registration for training.

For intervention paramedics who have not completed training, selection for interview will aim to represent only each ambulance service and base station setting (urban; rural), as further information about non trained paramedics is not captured during the study processes.

According to availability, focus groups or individual interviews (in person or by telephone) will be conducted. Paramedics taking part in both the internal pilot trial (up to 20) and main trial (up to 60) will be included. Individual paramedics may be invited for interview on more than one occasion. For intervention group paramedics who have completed training, interviews will focus on understanding views about delivery of the study intervention and the training process. For intervention group paramedics who have not completed training, interviews will focus (as appropriate) on why training has not been undertaken and views on why they may or may not undertake training if the intervention was part of standard clinical care.

Intervention group paramedics were originally made aware of the possibility of being invited for interview during the PASTA online training session. In order to make untrained intervention paramedics aware of the possibility of being interviewed, a study newsletter will be issued explaining that any paramedic allocated to the intervention group may be invited to take part.

Thereafter, at agreed time intervals during the trial recruitment period the researcher conducting the interviews will identify paramedics to be invited for interview. The researcher will subsequently contact the identified paramedics to discuss the interview and provide an information sheet. Having determined who is willing to take part, arrangements will be made for focus groups or individual interview (in person or by telephone), according to local availability. In advance of the interview, paramedics will be receive a consent form and be asked to return this by post, email or in person. However, due to the mobile nature of the paramedic workforce, it can be prohibitive to ask for return of completed forms prior to a telephone interview. If a telephone interview is planned and it is not practical to return the consent form by post or email in advance, verbal consent only will be used. This will be recorded by the researcher at the beginning of the interview. For face to face interviews or focus group, a written consent form will be completed.

4. Hospital professionals

Hospital professionals working in the ED and stroke service who have witnessed the PASTA pathway in use will be included. They will be selected according to their clinical role (doctor, nurse, radiographer); local patient admission route (via ED or direct to stroke unit); and the local thrombolysis assessment approach (in person; telemedicine). According to availability, focus groups or individual interviews (in person or by telephone) will be conducted. Hospital professionals experiencing both the internal pilot trial (up to 15) and main trial (up to 50) will be included. The number of hospital sites where professionals will be identified for invitation will be determined during the trial according to parameters such as number of PASTA pathway's witnessed and stroke service design. Individual professionals may be invited for interview on more than one occasion.

At agreed time intervals during the trial recruitment period, the researcher conducting the interviews will make contact with hospital site research support staff and ask them to: i) identify professionals who have witnessed the PASTA pathway, ii) provide these staff with an interview information sheet and iii) ascertain which staff are willing to take part in an interview. Having determined who is willing to take part, the research support staff and researcher conducting the interviews will work together to make arrangements for focus groups or individual interviews (in person or by telephone), according to local availability. In advance of the interview, hospital professionals will receive a consent form and be asked to return this to the researcher conducting the interview by post, email or in person.

Data collection

Separate patient and professional interview topic guides will be used to facilitate discussion. Data collection and analysis will occur concurrently to allow for issues or themes identified in earlier interviews to be explored in more depth in subsequent interviews.

Data preparation and analysis

All interviews will be digitally recorded and transcribed verbatim. In line with data protection legislation and research governance frameworks, all information pertaining to individuals/places will be anonymised. The qualitative analysis will adopt a constructivist grounded theory approach[36]. Open, then focused coding, will be undertaken and emergent codes from the analysis of this stage will be presented to the wider research team. A suitable software package (e.g. NVivo) will be used to facilitate data analysis management.

22. Ethics and regulatory issues

The study sponsor is Newcastle upon Tyne Hospitals NHS Foundation Trust. The study will be conducted in accordance with Research Governance Framework for Health and Social Care. Ethical and NHS Trust approvals will be sought. The study coordinating centre will require a written copy of local approval documentation before initiating each participating site and accepting participants into the study.

23. Confidentiality

Personal data will be regarded as strictly confidential. Original paper case record forms containing study data will be stored in the investigator site file at each research site. All study files will be securely stored and access restricted to staff involved in the study. NHS research support staff at sites will enter data from paper forms onto a secure web-based electronic database run and maintained by Newcastle University. Data will be entered using pseudo-anonymised participant identification codes and excepting for the qualitative interview study where specific consent has been obtained for contact detail to be given to the qualitative researcher, no identifiable information will be transferred out of the local site. Access to the database will be password protected and limited to staff at research sites or Newcastle University who are involved in the study.

The study will comply with the Data Protection Act 1998, and Caldicott Guardian approval for use of data will be sought in line with local requirements. All trial documentation will be retained for future audit and inspection in line with the sponsor policies.

24. Trial monitoring, quality control and quality assurance

The Chief Investigator will have overall responsibility for study conduct. The Principal Investigators will be responsible for the day-to-day study conduct at their individual sites.
The trial will be managed by a co-ordinating centre based at Newcastle University who will provide day-to-day support for the sites and provide training through investigator meetings, site initiation visits and routine monitoring visits. A Trial Management Group (TMG) will be convened and meet regularly during the study.

Quality control will be maintained through adherence to Newcastle Biomedicine Clinical Research Platform SOPs, the study protocol and research governance regulations. General monitoring of study conduct and data collected will be performed by a combination of central review and site monitoring visits. The main areas of focus will include consent, serious adverse events and essential documents in study files. All monitoring findings will be reported and followed up with the appropriate persons in a timely manner.

The study may be subject to inspection and audit by Newcastle upon Tyne Hospitals NHS Foundation Trust under their remit as sponsor.

A Trial Steering Committee will be convened to provide oversight of the trial. This will comprise of an independent Chair, two other independent members, a patient representative, and the Chief Investigator. Representatives from NIHR and the study sponsor will be invited to attend TSC meetings. The TSC will agree a charter of operation and meet at least annually during the study.

An independent Data Monitoring and Ethics Committee (DMEC) will be established. It will comprise of 4 members including expert healthcare professionals and a statistician. Only the

DMEC will have access to unblinded outcome data before the trial ends. The DMEC will agree a charter of operation and meet at least annually during the study.

25. Indemnity

Ambulance and Hospital NHS Trusts participating in the study have liability for clinical negligence that harms individuals toward whom they have a duty of care. NHS indemnity covers NHS staff and academic staff with honorary contracts conducting the trial for potential liability in respect of negligent harm arising from the conduct of the study. Newcastle upon Tyne Hospitals NHS Foundation Trust is the Sponsor and through the Sponsor, NHS indemnity is provided in respect of potential liability and negligent harm arising from study management. Indemnity in respect of potential liability arising from negligent harm related to study design is provided by NHS schemes for those protocol authors who have their substantive contracts of employment with the NHS and by Newcastle University Insurance schemes for those protocol authors who have their substantive contract of employment with the university. This is a non-commercial study and there are no arrangements for non-negligent compensation.

26. Funding

This study is funded by the National Institute for Health Research (NIHR) within the Programme Grant for Applied Research Programme entitled “Promoting Effective And Rapid Stroke Care” (RP-PG-1211-20012).

27. Dissemination of results

The data will be the property of the Chief Investigator and Co-Investigator(s). Publication will be the responsibility of the Chief Investigator.

The study will be presented at national and international conferences, and reported peer reviewed journals. Reports will be written for the study funder, sponsor and regulatory bodies. Anonymised data will be provided to research databases as requested (e.g. the Cochrane Collaboration) to enable future meta-analyses.

28. References

1. Feigin VL, Forouzanfar MH, Krishnamurthi R, Mensah GA, Connor M, Bennett DA, Moran AE, Sacco RL, Anderson L, Truelsen T, et al: **Global and regional burden of stroke during 1990-2010: findings from the Global Burden of Disease Study 2010.** *Lancet* 2014, **383:**245-254.
2. National Audit Office: **Reducing brain damage: faster access to better stroke care**. London; 2005.
3. Wardlaw JM, Murray V, Berge E, Del Zoppo GJ: **Thrombolysis for acute ischaemic stroke.** *The Cochrane database of systematic reviews* 2009**:**CD000213.
4. National Institute for Health and Care Excellence: **Alteplase for treating acute ischaemic stroke (review of technology appraisal guidance 122)**. NICE technology appraisal guidance 264; 2012.
5. Clinical Effectiveness and Evaluation Unit Royal College of Physicians: **The Sentinel Stroke National Audit Programme (SSNAP)**. https://www.rcplondon.ac.uk/projects/ssnap-clinical-audit.
6. Lorenzano S, Ahmed N, Tatlisumak T, Gomis M, Davalos A, Mikulik R, Sevcik P, Ollikainen J, Wahlgren N, Toni D, SITS Investigators: **Within-day and weekly variations of thrombolysis in acute ischemic stroke: results from safe implementation of treatments in stroke-international stroke thrombolysis register.** *Stroke* 2014, **45:**176-184.
7. Jauch EC, Saver JL, Adams HP, Jr., Bruno A, Connors JJ, Demaerschalk BM, Khatri P, McMullan PW, Jr., Qureshi AI, Rosenfield K, et al: **Guidelines for the early management of patients with acute ischemic stroke: a guideline for healthcare professionals from the American Heart Association/American Stroke Association.** *Stroke* 2013, **44:**870-947.
8. Intercollegiate Stroke Working Party: **National clinical guideline for stroke 4th edition**. Royal College of Physicians; 2012.
9. Harbison J, Hossain O, Jenkinson D, Davis J, Louw SJ, Ford GA: **Diagnostic accuracy of stroke referrals from primary care, emergency room physicians, and ambulance staff using the face arm speech test.** *Stroke* 2003, **34:**71-76.
10. Patel MD, Rose KM, O'Brien EC, Rosamond WD: **Prehospital notification by emergency medical services reduces delays in stroke evaluation: findings from the North Carolina stroke care collaborative.** *Stroke* 2011, **42:**2263-2268.
11. Mosley I, Nicol M, Donnan G, Patrick I, Kerr F, Dewey H: **The impact of ambulance practice on acute stroke care.** *Stroke* 2007, **38:**2765-2770.
12. Alberti G: **Emergency care ten years on: reforming emergency care**. Department of Health; 2007.
13. Berglund A, Svensson L, Sjostrand C, von Arbin M, von Euler M, Wahlgren N, Collaborators H, Engerstrom L, Hojeberg B, Kall TB, et al: **Higher prehospital priority level of stroke improves thrombolysis frequency and time to stroke unit: the Hyper Acute STroke Alarm (HASTA) study.** *Stroke* 2012, **43:**2666-2670.
14. Ebinger M, Winter B, Wendt M, Weber JE, Waldschmidt C, Rozanski M, Kunz A, Koch P, Kellner PA, Gierhake D, et al: **Effect of the use of ambulance-based thrombolysis on time to thrombolysis in acute ischemic stroke: a randomized clinical trial.** *JAMA* 2014, **311:**1622-1631.
15. Liman TG, Winter B, Waldschmidt C, Zerbe N, Hufnagl P, Audebert HJ, Endres M: **Telestroke ambulances in prehospital stroke management: concept and pilot feasibility study.** *Stroke* 2012, **43:**2086-2090.
16. Bergrath S, Reich A, Rossaint R, Rortgen D, Gerber J, Fischermann H, Beckers SK, Brokmann JC, Schulz JB, Leber C, et al: **Feasibility of prehospital teleconsultation in acute stroke--a pilot study in clinical routine.** *PLoS One* 2012, **7:**e36796.
17. McMeekin P, Gray J, Ford GA, Rodgers H, Price CI: **Modelling the efficiency of local versus central provision of intravenous thrombolysis after acute ischemic stroke.** *Stroke* 2013, **44:**3114-3119.

18. Choi B, Tsai D, McGillivray CG, Amedee C, Sarafin JA, Silver B: **Hospital-directed feedback to Emergency Medical Services improves prehospital performance.** *Stroke* 2014, **45:**2137-2140.
19. Iedema R, Ball C, Daly B, Young J, Green T, Middleton PM, Foster-Curry C, Jones M, Hoy S, Comerford D: **Design and trial of a new ambulance-to-emergency department handover protocol: 'IMIST-AMBO'.** *BMJ Quality and Safety* 2012, **21:**627-633.
20. Ko HC, Turner TJ, Finnigan MA: **Systematic review of safety checklists for use by medical care teams in acute hospital settings--limited evidence of effectiveness.** *BMC health services research* 2011, **11:**211.
21. Brott T, Adams HP, Jr., Olinger CP, Marler JR, Barsan WG, Biller J, Spilker J, Holleran R, Eberle R, Hertzberg V, et al.: **Measurements of acute cerebral infarction: a clinical examination scale.** *Stroke* 1989, **20:**864-870.
22. van Swieten JC, Koudstaal PJ, Visser MC, Schouten HJ, van Gijn J: **Interobserver agreement for the assessment of handicap in stroke patients.** *Stroke* 1988, **19:**604-607.
23. NHS Commissioning Board Knowledge and Intelligence Branch: **Everyone Counts: Planning for Patients 2013/14: Technical Definitions**. NHS Commissioning Board; 2012.
24. Wahlgren N, Ahmed N, Davalos A, Ford GA, Grond M, Hacke W, Hennerici MG, Kaste M, Kuelkens S, Larrue V, et al: **Thrombolysis with alteplase for acute ischaemic stroke in the Safe Implementation of Thrombolysis in Stroke-Monitoring Study (SITS-MOST): an observational study.** *Lancet* 2007, **369:**275-282.
25. Bruno A, Akinwuntan AE, Lin C, Close B, Davis K, Baute V, Aryal T, Brooks D, Hess DC, Switzer JA, Nichols FT: **Simplified modified rankin scale questionnaire: reproducibility over the telephone and validation with quality of life.** *Stroke* 2011, **42:**2276-2279.
26. Wardlaw JM, Sandercock PA, Warlow CP, Lindley RI: **Trials of thrombolysis in acute ischemic stroke: does the choice of primary outcome measure really matter?** *Stroke* 2000, **31:**1133-1135.
27. Lovett JK, Coull AJ, Rothwell PM: **Early risk of recurrence by subtype of ischemic stroke in population-based incidence studies.** *Neurology* 2004, **62:**569-573.
28. The CONSORT statement: http://www.consort-statement.org/.
29. McMeekin P, Flynn D, Ford GA, Rodgers H, Gray J, Thompson RG: **Development of a decision analytic model to support decision making and risk communication about thrombolytic treatment.[Erratum appears in BMC Med Inform Decis Mak. 2016;16:4; PMID: 26775145].** *BMC Medical Informatics & Decision Making*, **15:**90.
30. Rivero-Arias O, Ouellet M, Gray A, Wolstenholme J, Rothwell PM, Luengo-Fernandez R: **Mapping the modified Rankin scale (mRS) measurement into the generic EuroQol (EQ-5D) health outcome.** *Med Decis Making* 2010, **30:**341-354.
31. Manca A, Hawkins N, Sculpher MJ: **Estimating mean QALYs in trial-based cost-effectiveness analysis: the importance of controlling for baseline utility.** *Health Economics* 2005, **14:**487-496.
32. Fenwick E, Claxton K, Sculpher M: **Representing uncertainty: the role of cost-effectiveness acceptability curves.** *Health Economics* 2001, **10:**779-787.
33. Manca A, Palmer S: **Handling missing data in patient-level cost-effectiveness analysis alongside randomised clinical trials.** *Applied Health Economics & Health Policy* 2005, **4:**65-75.
34. Siebert U, Alagoz O, Bayoumi AM, Jahn B, Owens DK, Cohen DJ, Kuntz KM, Force I-SMGRPT: **State-transition modeling: a report of the ISPOR-SMDM Modeling Good Research Practices Task Force--3.** *Value in Health* 2012, **15:**812-820.
35. Felli JC, Hazen GB: **Sensitivity analysis and the expected value of perfect information.[Erratum appears in Med Decis Making. 2003 Jan-Feb;23(1):97.], [Erratum appears in Med Decis Making 2001 May-Jun;21(3):254].** *Medical Decision Making* 1998, **18:**95-109.
36. Charmaz K: **Constructing Grounded Theory: A Practical Guide through Qualitative Analysis***.* London: SAGE Publications Ltd; 2006.

Appendices

Appendix A: Summary diagram

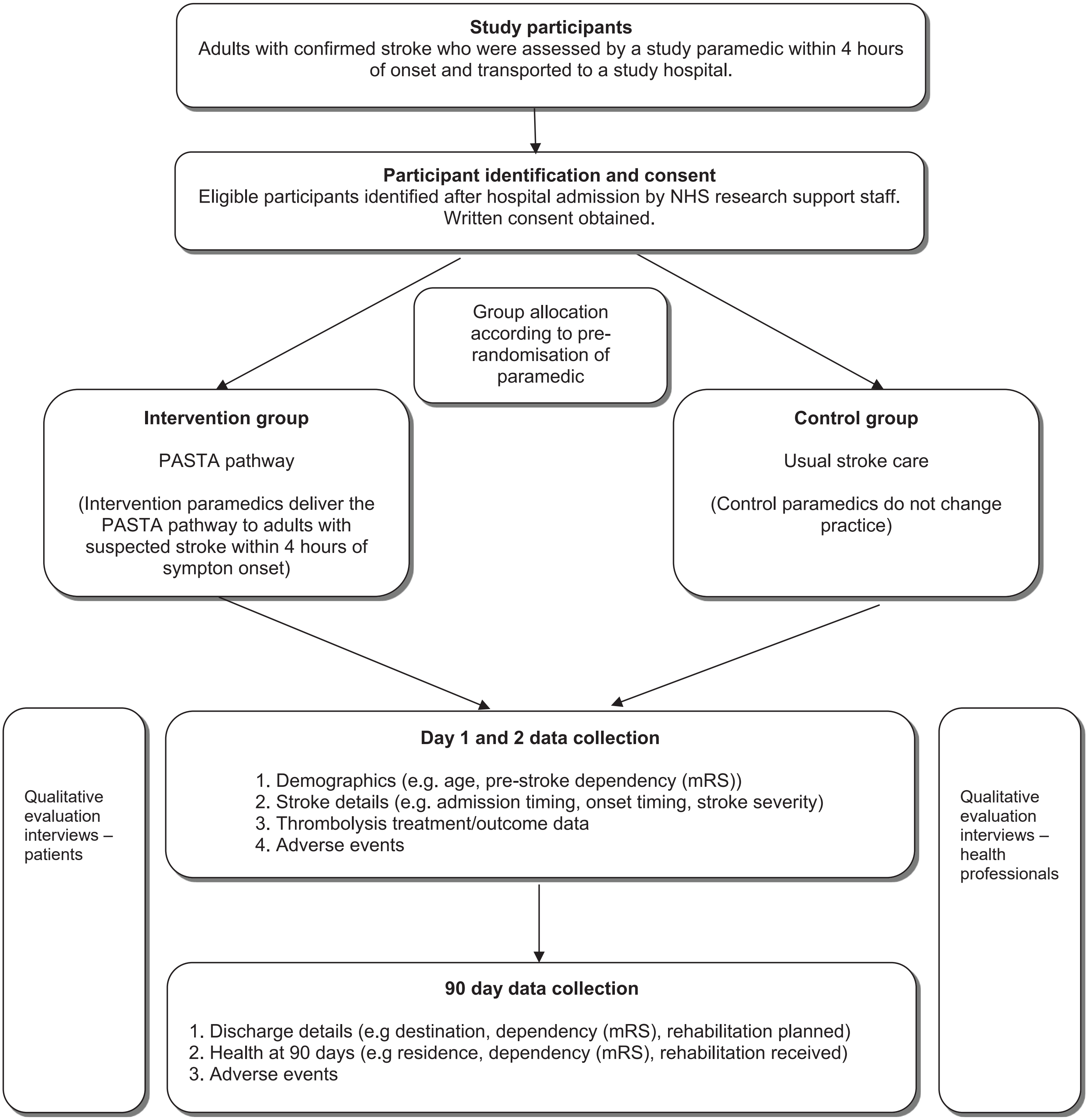

Appendix B. Modified Rankin Scale

**0**	No symptoms at all
**1**	No significant disability despite symptoms: able to carry out all usual duties and activities
**2**	Slight disability: unable to carry out all previous activities but able to look after own affairs without assistance
**3**	Moderate disability: requiring some help, but able to walk without assistance
**4**	Moderately severe disability: unable to walk without assistance, and unable to attend to own bodily needs without assistance
**5**	Severe disability: bedridden, incontinent, and requiring constant nursing care and attention
