# Supplementary material for: Effect of an Enhanced Paramedic Acute Stroke Treatment Assessment on Thrombolysis Delivery During Emergency Stroke Care: A Cluster Randomized Clinical Trial
Source: JAMA Neurol. 2020 Apr 13;77(7):1–9. doi: 10.1001/jamaneurol.2020.0611 (PMC7154959; doi:10.1001/jamaneurol.2020.0611)
Supplement: Supplement 3. — Data Sharing Statement. [file jamaneurol-77-840-s003.pdf]

Price CI. Effect of an enhanced Paramedic Acute Stroke Treatment Assessment on thrombolysis delivery during emergency stroke care: a cluster randomized controlled trial. *JAMA Neurol.* Published online April 13, 2020. doi:10.1001/jamaneurol.2020.0611

Data Sharing Statement

Data
Data available: No
